# Construction and Validation of Protein Expression-related Prognostic Models in Clear Cell Renal Cell Carcinoma

**DOI:** 10.7150/jca.81915

**Published:** 2023-03-27

**Authors:** Xuzhan Ma, Libin Sun

**Affiliations:** Department of Urology, Affiliated First Hospital of Shanxi Medical University, Taiyuan, Shanxi, China.

**Keywords:** renal clear cell carcinoma, protein prognostic model, immunohistochemistry, potential drug treatment, biological information

## Abstract

**Objective:** To construct a prognostic evaluation model for clear cell renal cell carcinoma (ccRCC) patients using bioinformatics method and to screen potential drugs for ccRCC**.**

**Methods:** ccRCC RNA sequencing data, clinical data, and protein expression data were downloaded from the TCGA database. Univariate Cox and Lasso regression analyses were performed on the combined data to screen out the proteins related to the prognosis, and they were included in a multivariate Cox proportional hazard model. The patients were divided into high and low-risk groups for a survival difference analysis. The predictive power of the model was evaluated on the basis of overall survival, progression-free survival, independent prognostic, clinically relevant receiver operating characteristic (ROC) curve, C-index, principal component, and clinical data statistics analyses. GSEA enrichment and immune function correlation analyses were performed. The samples were divided into different subtypes based on the expression of the risk proteins, and survival analysis of the subtypes was performed. The risk-related protein and RNA sequencing data were analyzed to screen out sensitive drugs with significant differences between the high and low-risk groups.

**Results:** A total of 469 ccRCC-related proteins were screened, of which 13 proteins with independent prognostic significance were screened by univariate Cox, Lasso, and multivariate Cox regression analyses to construct the prognostic model. The sensitivity and accuracy of the model in predicting the survival of patients with ccRCC were high (1 year: 0.811, 3 years: 0.783, 5 years: 0.777). The 13 proteins were closely related to immunity, and the model proteins were different between kidney and tumor tissues according to the HPA database. The samples were divided into three subtypes, and there were obvious clinical characteristics of the three subtypes in the grade and T, N and M stages. According to the IC50 values, CGP-60474, vinorelbine, doxorubicin, etoposide, FTI-277, JQ12, OSU-03012, pyrimethamine, and other drugs were more sensitive in the high-risk group.

**Conclusions:** A prognostic model of protein expression in ccRCC was successfully constructed, which had good predictive ability for the prognosis of ccRCC patients. The ccRCC-related proteins in the model can be used as targets for studying the pathogenesis and targeted therapy.

## Introduction

Renal cell carcinoma (RCC), also known as renal adenocarcinoma or renal carcinoma, originates from the urinary tubular epithelial system of the renal parenchyma accounts for 80-90% of malignant renal tumors^1^. Clear cell renal cell carcinoma (ccRCC) accounts for about 70-80% of renal cancers. More patients with renal cell carcinoma have been detected clinically with continuous improvement in the diagnosis, increasing awareness of medical care, and the gradual extension of the national average life expectancy^2^, ^3^. The incidence of renal cell carcinoma in China has shown a gradual upward trend^4^.

The main treatment for ccRCC is surgery^5^. However, because the symptoms of early ccRCC are not obvious at the time of the diagnosis, and the disease is already in the advanced stage or has metastasized, the opportunity for surgery is missed^6^-^8^. The effect of radiotherapy and chemotherapy is poor for these patients. Molecular targeted therapy can significantly improve the objective response rate of patients with metastatic ccRCC, prolong progression-free survival (PFS) and overall survival (OS), and significantly prolong the life of patients compared with traditional cytokine therapy^9^-^11^. However, the response time to treatment and survival benefits vary greatly among patients^8^.

With the development of sequencing technology, many studies have shown that although a variety of mRNA, miRNA, lncRNA, ceRNA and other genetic markers have a good predictive ability on the prognosis of ccRCC, there is still a lack of specific and sensitive biomarkers for diagnosis and treatment. Protein expression plays a key role in different stages of tumorigenesis, but no protein prognostic model has been studied in ccRCC. Protein-level research is more advantageous than RNA-level research for clinical application; therefore, this study proposed using protein levels to analyze ccRCC. To provide important reference data for the accuracy of clinical diagnosis, treatment and prognosis of ccRCC, a prognosis model of multiple protein-bound prognosis will be constructed from the perspective of protein.

## Materials and Methods

### Data download and sample collation

ccRCC RNA sequencing, clinical, and protein expression data were downloaded from the TCGA database (https://portal.gdc.cancer.gov). The control and patient clinical data included age, gender, survival time and survival state, tumor classification, and T, N, and M stages. Perl software was used for data collation, identifying cases with complete clinicopathological information of the clincal samples, converting the IDs of the RNA sequencing data.

### Construction of the prognostic model

The limma and impute packages in R-studio software (The R Foundation for Statistical Computing, Vienna, Austria) were used to merge the protein expression data with the clinical data (survival time and survival status). The survival, caret, glmnet, survminer and timeROC packages were used to perform the univariate Cox regression analysis (filtering criteria: P < 0.05) on the combined data to screen for proteins related to the prognosis of patients with ccRCC. Lasso regression analysis was performed to reduce the overfitting of the data and to screen the key proteins. The Lasso regression used cross-validation to select the parameters, and the Lasso regression coefficient spectrum was drawn. Finally, multivariate Cox regression analysis was performed to establish the risk protein model for the prognosis of ccRCC, which was displayed in the form of a nomogram. The risk protein model was constructed based on the multivariate Cox regression analysis, and the risk score equation was: Risk score = *∑coefficient_i_ × EXP (protein)_I_*. The data were randomly divided into two groups (training and test groups), and the group samples were divided into a high- or low-risk group according to the median risk score, respectively.

### Evaluation and clinical value of the prognostic model

R-studio software was used for the statistical analysis of the training group and the test group. The dplyr, ggplot2, and ggrepel packages were used to analyze the results of the univariate Cox analysis (defined significance: P < 0.05, HR > 1 was high risk, HR < 1 was low risk), and a volcano plot was drawn. The corrplot, circlize, ggalluvial, ggplot2, and dplyr packages were used for the co-expression analysis of the risk protein data to clarify the correlation between the proteins in the model and the co-expression relationship between the proteins in the model and other proteins (Cor value was set to 0.4). Principal component analysis (PCA) analysis was performed using the limma and scatterplot3d packages to verify whether the proteins involved in constructing the model could distinguish patients in the high and low-risk groups. The survival and survminer packages were used to analyze OS and PFS of the risk protein data. According to the expression levels of the proteins in the model, the samples were divided into high and low-expressing groups, and the survival analysis of the proteins in the model was performed. The survival, survminer, and timeROC packages were used to perform the independent prognostic analysis of the risk protein and the clinically relevant data, and a receiver operating characteristic (ROC) curve analysis was performed. The dplyr, survival, rms, and pec packages were used to perform the C-index analysis of the risk gene data and the clinically relevant data. The regplot, survival, and rms packages were used to draw a nomogram between the risk protein data and the clinically relevant data to predict the survival of patients with ccRCC through the nomogram. The survival, survminer, limma, and ggpubr packages were used to verify the clinical grouping model and the clinical correlation analysis of the risk protein data, and the clinically relevant data were used to verify whether the constructed model was suitable for patients in the different clinical groups. The proteins and risk scores in the model were analyzed to detect significant differences between the clinical groups.

### Enrichment analysis of the risk proteins

The limma, clusterProfiler, org.Hs.eg.db, and enrichplot packages were used to perform the GO enrichment and KEGG analyses on the RNA sequencing and risk protein data, respectively. The results were visualized, and the top five enriched pathways with high and low risk were plotted.

### Immune-related functional analysis of the risk proteins

The limma package in R-studio software was used to sort the RNA sequencing data and CIBERSORT was run to obtain the immune cell infiltration results. The ggpubr and limma packages were used to analyze the differences in immune cell infiltration and the risk protein data. Furthermore, a radar map was drawn using the fmsb package. The National Library of Medicine (https://www.ncbi.nlm.nih.gov) was accessed to identify the standard names of the proteins in the model, and the model proteins were analyzed by immunohistochemistry using the HPA database. RNA sequencing data uploaded to TIDE database (http://tide.dfci.harvard.edu/), get the TIDE score, and then through limma, ggpubr package to analyze risk protein data, clear TIDE score between high-risk and low-risk groups if there is a difference.

### Survival analysis and clinical correlation analysis of the sample types

The limma and ConsensusClusterPlus packages were used to classify the samples according to the expression of the model proteins, and the samples were divided into different subtypes. The survival and survminer packages were used to analyze the survival of the subtypes to determine whether there was a difference in survival time between the subtypes. Finally, the ggplot2 package was used to analyze the clinical correlation between the subtypes and the clinical data (age, gender, grade, and T, N, and M stages).

### Screening of potential drugs for ccRCC

The limma, ggpubr, pRRophetic, and ggplot2 packages were used to analyze the RNA sequencing and risk protein data, and the drugs with significant differences between the high and low-risk groups were screened. The screening criterion was P < 0.001.

## Results

### Construction of the prognostic model

**Figure 1:**
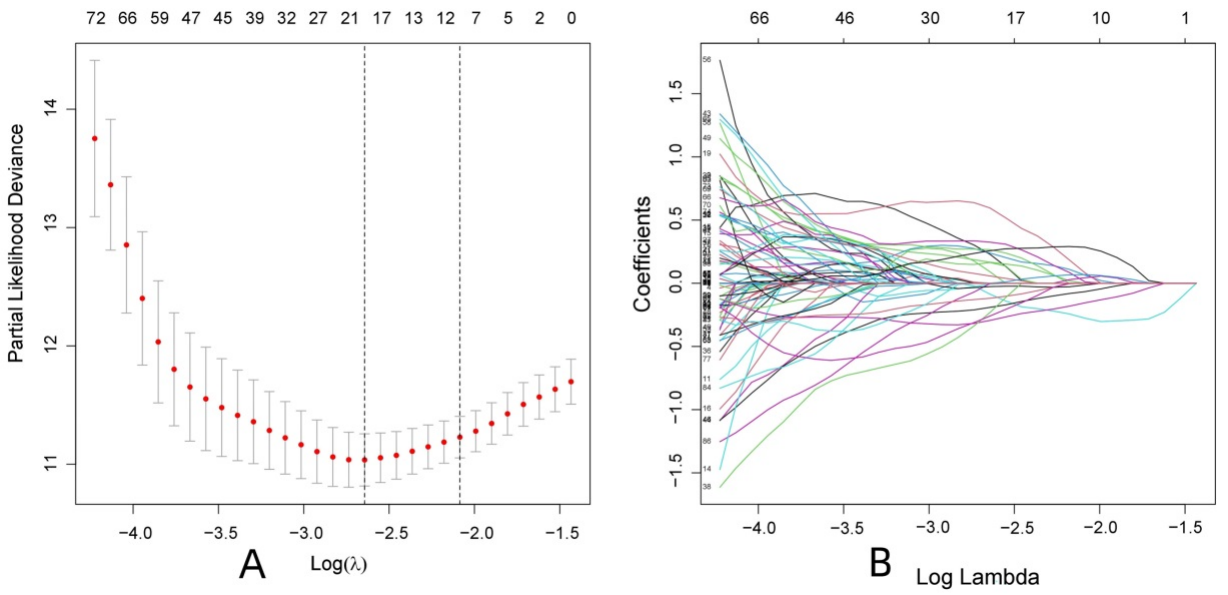
Screening for proteins with greater prognostic value in patients with ccRCC. (Lasso regression analysis)

R-studio software was used to combine the protein data related to ccRCC with the clinical data (survival time and survival status). First, 178 proteins related to the prognosis of patients with ccRCC were initially screened by univariate Cox regression analysis (P < 0.05), and Lasso regression analysis was used to reduce overfitting of the data. Nineteen proteins that were more valuable for the prognosis of ccRCC patients were screened (**Fig. 1 and Supplementary Table S1**). Finally, 13 proteins valuable for the prognosis were screened out through the multivariate Cox regression analysis (**Table 1**), and the prognostic model was constructed according to the risk scores of the 13 proteins. The samples were randomly divided into the training and test groups, and the samples of these groups were further divided into high and low-risk groups according to the median value of the risk score formula: (1.15731496517862 × MITF) + (0.650254982564728 × SHP2_pY542) + (0.377159976590908×IGFBP2) + (-0.445732648417367 × AMPKALPHA_pT172) + (0.714167488114541 × ACC1) + (-1.11263944527678 × P70S6K_pT389) + (1.33597513048865 × RRM2) + (1.40730568252638 × PEA15) + (-0.549016199086467 × MAPK_pT202Y204) + (-0.31455704279515 × UGT1A) + (-1.45077304547422 × BRAF_pS445) + (0.29021723435788 × 4EBP1_pt37t46) + (0.939738175853776 × Vinculin).

### Evaluation and clinical value of the prognostic models

**Figure 2:**
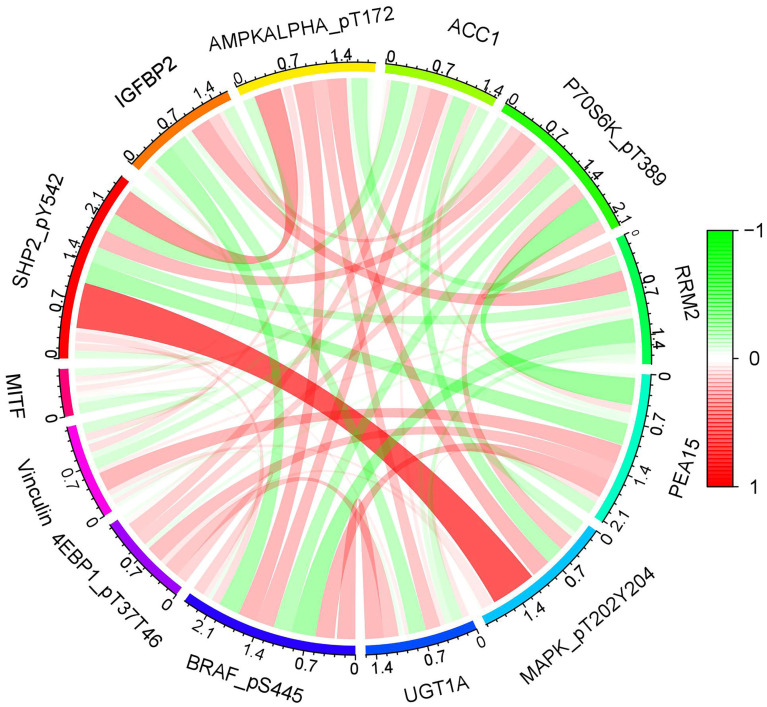
Co-expression analysis of model proteins. A: the correlation between each protein in the model. B: The correlation between the model proteins and other proteins.

**Figure 3:**
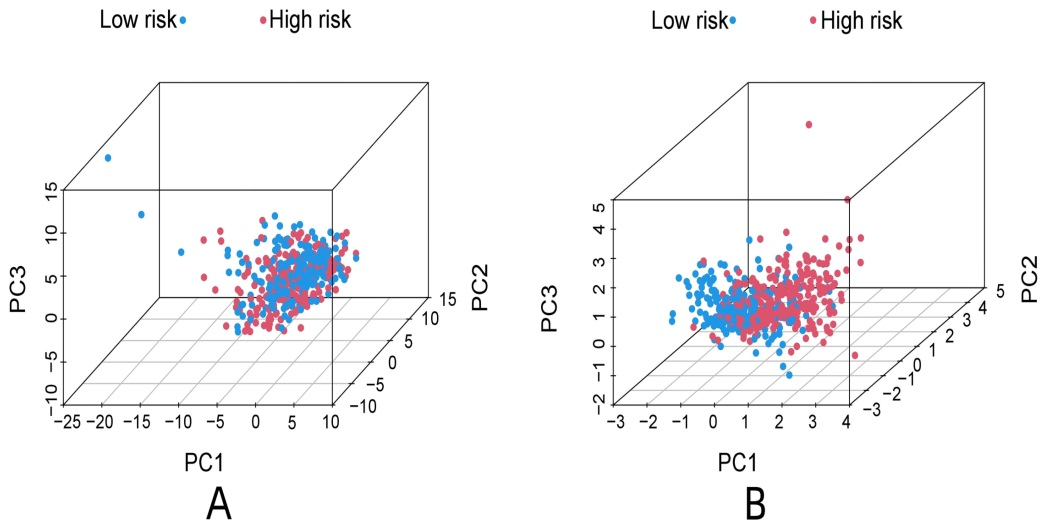
PCA analysis of all proteins associate to ccRCC (A), PCA analysis of model proteins (B).

**Figure 4:**
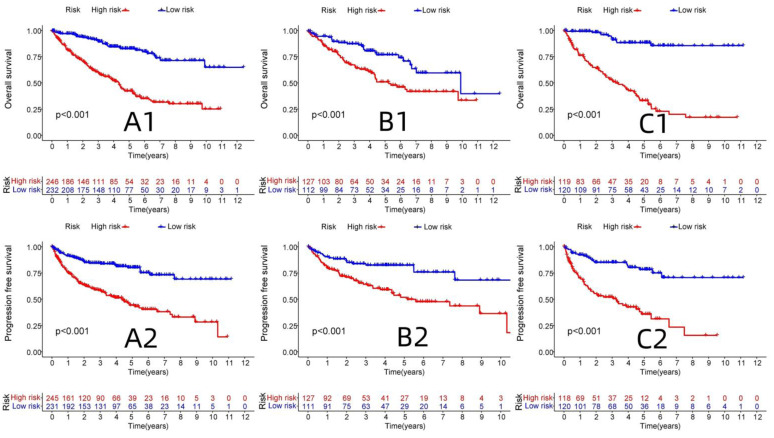
OS and PFS analysis between high and low risk groups in Train group and Test group. (A1: OS analysis of all proteins associate to ccRCC, B1: OS analysis of Test group, C1: OS analysis of Train group, A2: PFS analysis of all proteins associate to ccRCC, B2: PFS analysis of Test group, C2: PFS analysis of Train group)

**Figure 5:**
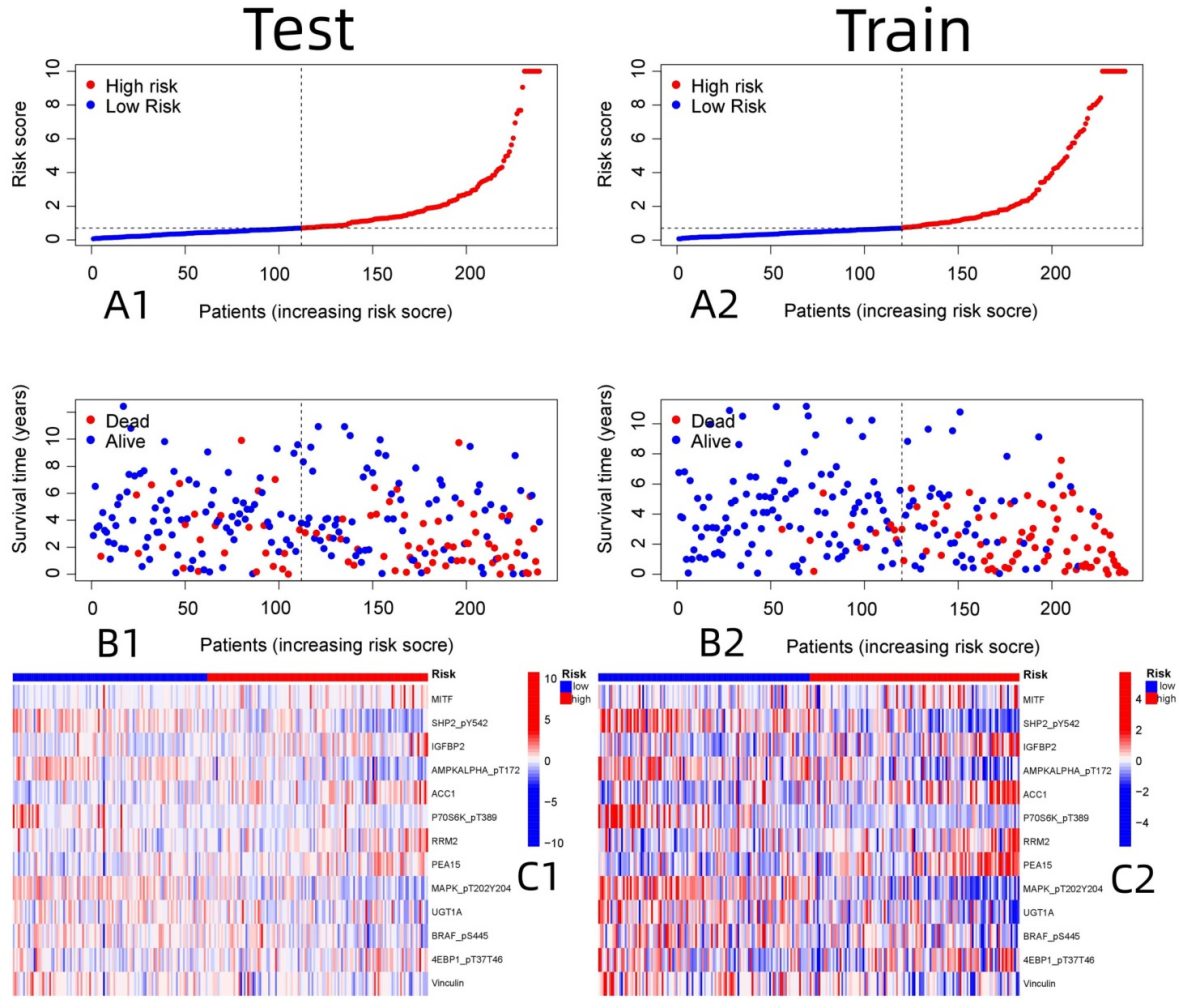
Patient risk score in relation to risk and survival, and the expression analysis of 13 model proteins in the high and low risk of Train and Test group. (A1: Patient risk score was associated with risk in the Test group, A2: Patient risk score was associated with risk in the Train group, B1: Patient risk score was associated with survival in the Test group, B2: Patient risk score was associated with survival in the Train group, C1: Expression of 13 model proteins screened in Test group, C2: Expression of 13 model proteins screened in Train group)

**Figure 6:**
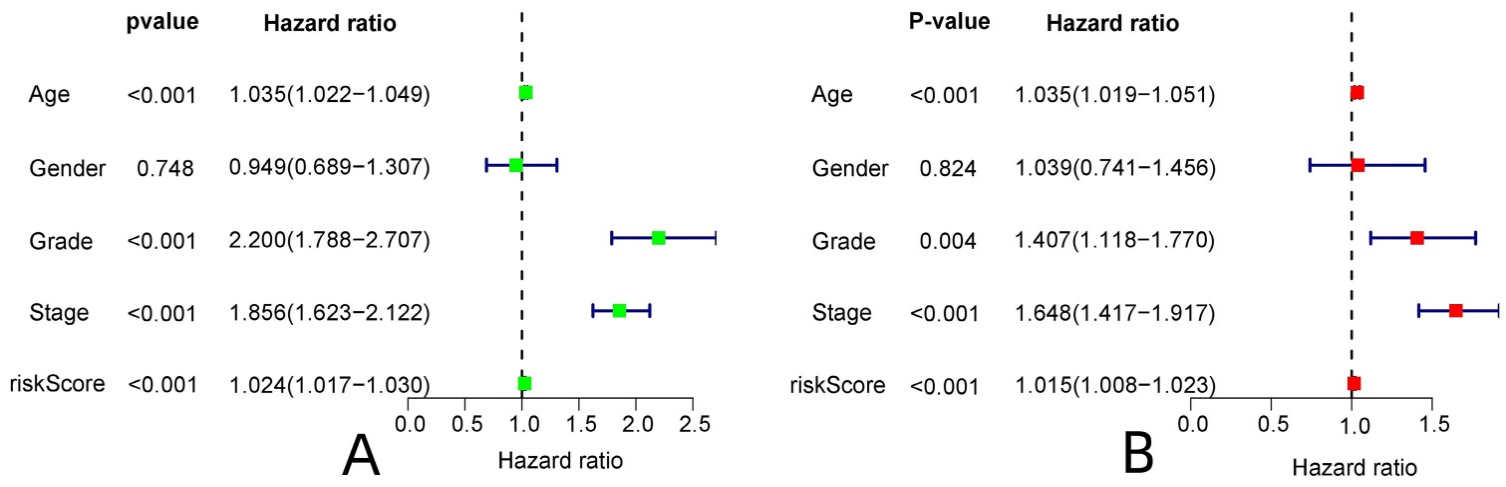
Independent prognostic analysis of the constructed model. (A: Univariate Cox analysis, B: Multivariate Cox analysis)

**Figure 7:**
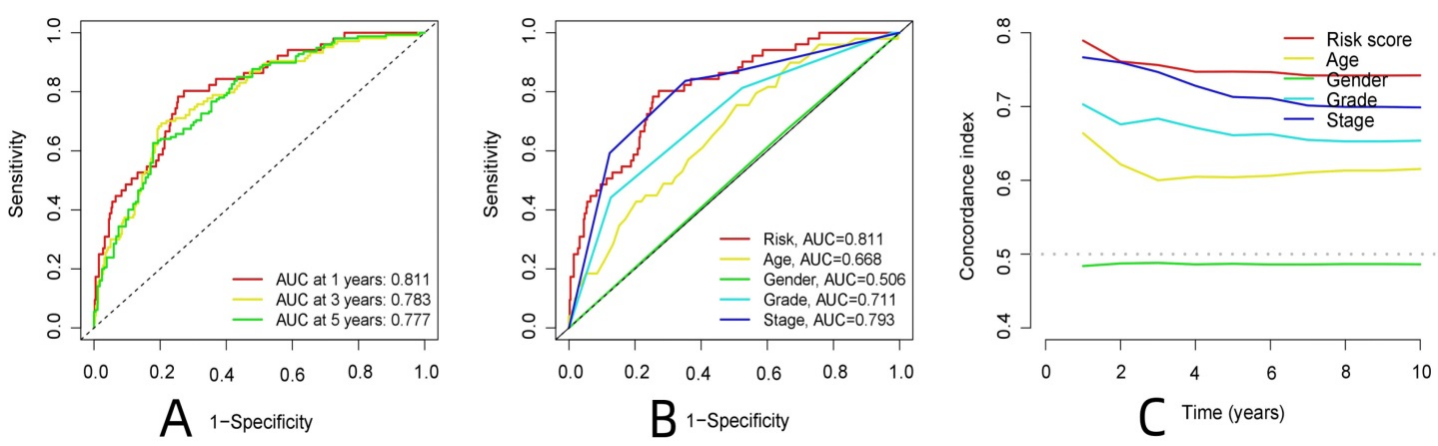
Analysis of the model to predict patient survival. (A: ROC curve of the constructed model, B: The ROC curve of the constructed model was analyzed jointly with clinical data, C: The C-index curve of the constructed model,)

**Figure 8:**
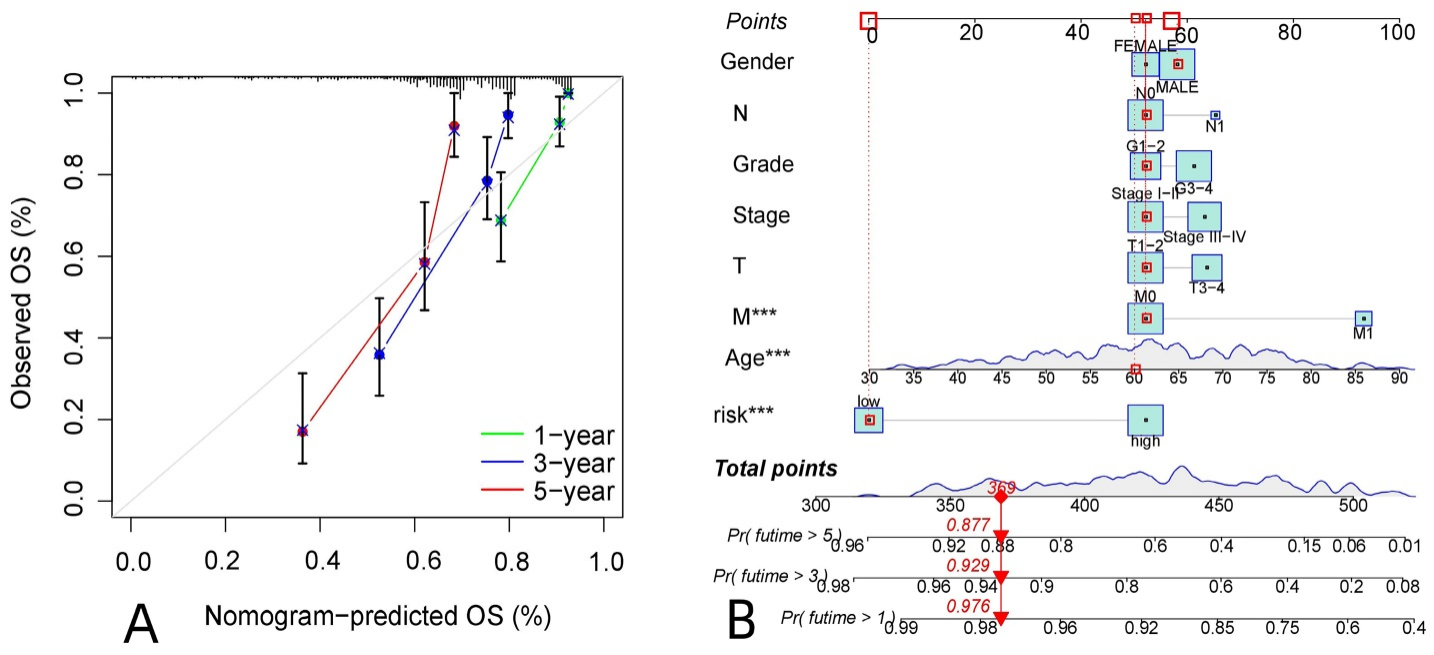
Prediction of survival in patients with ccRCC.A: The calibration plot of patients with ccRCC, B: The survival column chart of patients with ccRCC

**Figure 9:**
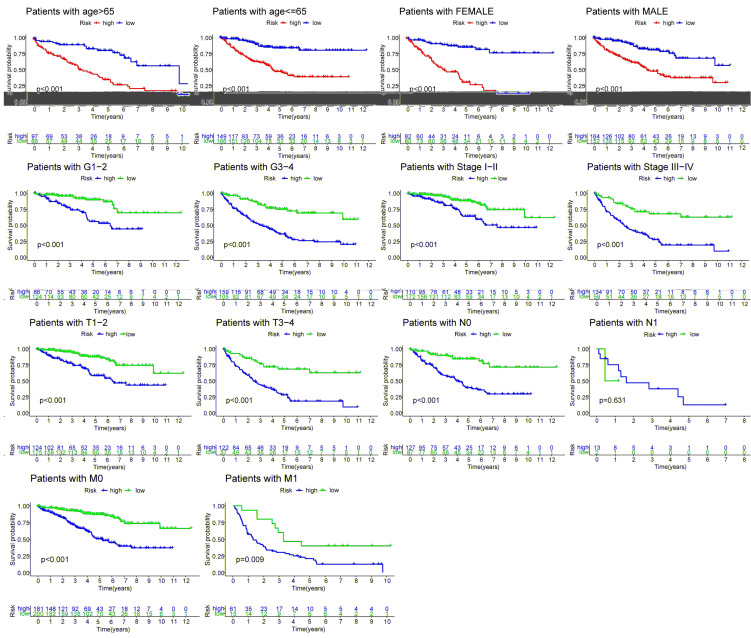
Correlation analysis between risk proteins and clinical relevant data

R-studio software was used for the clinical statistical analysis of the training and test groups. The results revealed no significant differences in any of the clinical traits between the training group and the test group, indicating no deviation in the clinical traits when the samples were randomly grouped (P > 0.05) (**Table 2**). Co-expression analysis was performed and a co-expression circle diagram was drawn to determine the correlation between each protein in the model. The correlation between the model protein and other proteins was determined (**Fig. 2 and Supplementary Table S2**). PCA analysis discriminated between the high and low-risk groups of the constructed model proteins, indicating that patients in the high and low-risk groups could be distinguished by the proteins in the model (**Fig. 3**). The OS and PFS analyses of the risk protein data showed significant differences in OS and PFS between the high and low-risk groups in the training and test groups. The OS and PFS of patients in the high-risk group were shorter than those in the low-risk group (**Fig. 4**). The grouping of the high and low-risk groups (the median of the risk score) was visualized with a risk curve by analyzing the risk protein data of the training and test groups. The survival state and heatmaps revealed that the death cases also increased with increased risk of the patients. MITF, IGFBP2, ACC1, RRM2, PEA15, and 4EBP1_pT37T46 were high-risk proteins in the training and test groups. SHP2_Py542, AMPKALPHA_pT172, P70S6K_pT389, MAPK_pT202Y204, UGT1A, BRAF_pS445, and Vinculin were identified as low-risk proteins (**Fig. 5**). The univariate and multivariate Cox analyses suggested that the constructed model could be used as an independent prognostic factor for the prognosis of patients with ccRCC, independent of other clinical traits (P < 0.001 for the risk score) (**Fig. 6**). The ROC curve showed that the sensitivity and accuracy of the model in predicting the survival of patients with ccRCC were high (AUC at 1 year: 0.811, AUC at 3 years: 0.783, AUC at 5 years: 0.777). The ROC curve combined with the clinically relevant data indicated that the constructed model predicted the survival of patients (AUC = 0.811) better than age, gender, grade, or stage (**Fig. 7A, B**). The constructed model predicted the survival of patients with high accuracy according to the C-index curve (**Fig. 7C**). The calibration plot showed that the predicted probability was consistent with the actual probability. The risk protein data were combined with the clinically relevant data to draw a survival nomogram for patients with ccRCC. The nomogram was used to score the patient's risk and predict survival rates at 1, 3, and 5 years. (**Fig. 8A, B**). The risk protein and clinically relevant data indicated significant differences in survival time between the high and low-risk groups for age > 65, age ≤ 65, gender, G1-2, G3-4, Stage I-II, Stage III-IV, T1-2, T3-4, M0, M1, and N0. This result shows that the constructed model was suitable for predicting survival in the clinical groups (**Fig. 9**).

Taken together, these results indicate that the prediction model based on 13 ccRCC-related risk proteins was significantly superior to clinical factors, such as age, gender, tumor grade, and stage in predicting the prognosis of patients with ccRCC, and the risk score was significantly correlated with the progression of ccRCC.

### Enrichment analysis of the risk proteins

**Figure 10:**
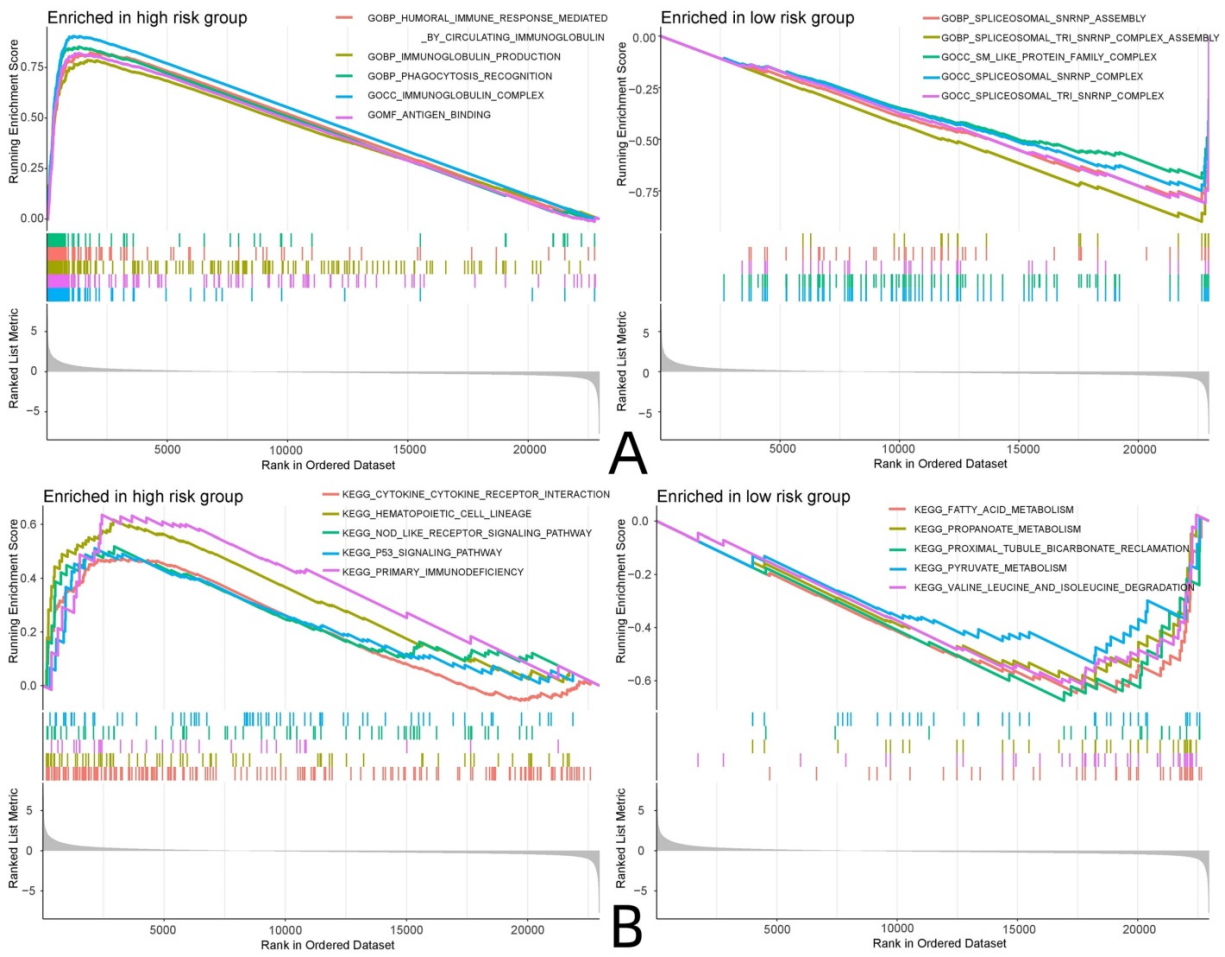
GSEA enrichment analysis. (A: GO enrichment analysis, B: KEGG enrichment analysis)

GO and KEGG enrichment analyses were performed on the RNA sequencing and risk protein data, and the enriched pathways in the high and low-risk groups were analyzed. The GO analysis showed that the risk proteins in the high-risk group were mainly enriched in GBP-humoral immune responses mediated by circulating immune factors, GBP-immunoglobulin production, GOBP-phagocytosis recognition, the GOCC-immunoglobulin complex, GOMF-antigen binding, and other related pathways. The risk proteins in the low-risk group were mainly enriched in the GOBP-spliceosomal snRNP assembly, the GOBP-spliceosomal TRI snRNP complex assembly, the GOCC-SM-like protein family complex, the GOCC-spliceosomal snRNP complex, the GOCC-spliceosomal TRI snRNP complex, and other related pathways. KEGG enrichment analysis showed that the risk proteins in the high-risk group were mainly enriched in cytokine-cytokine receptor interactions, hematopoietic cell lineage, the nod-like receptor signaling pathway, the p53 signaling pathway, and primary immunodeficiency and other related pathways. The risk proteins in the low-risk group were mainly enriched in fatty acid metabolism, propanoate metabolism, reclamation of bicarbonate in the proximal tubule, pyruvate metabolism, valine leucine and isoleucine degradation, and other related pathways (**Fig. 10**).

### Immune-related functional analysis of the risk proteins

**Figure 11:**
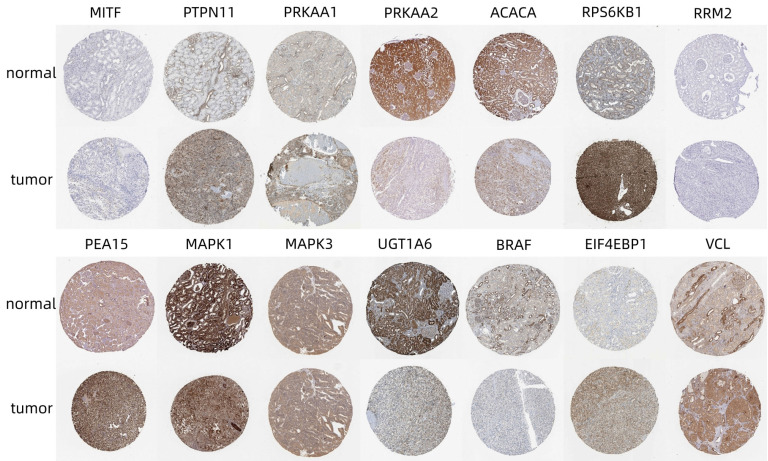
Immunohistochemical analysis of model proteins

The National Library of Medicine (https://www.ncbi.nlm.nih.gov) was accessed to identify the standard names of the proteins in the model, and the model proteins were analyzed by immunohistochemistry using the HPA database (**Table 1**). MITF expression was moderately positive in normal renal tissues (no glomerular cells were detected, and moderate staining was found in the renal collecting duct and distal tubules), but weakly positive in tumor tissues using the CAB002578 antibody. PTPN11 was moderately positive in most normal renal tissues according to CAB005377 staining (25-75% of glomerular and tubular cells were moderately stained) and was moderately positive in > 75% of the tumor tissues. PRKAA1 expression was moderately positive in normal renal tissues (moderate in renal tubular cells) and weakly positive in tumor tissues according to CAB005050 staining. PRKAA2 was strongly positive in most renal tissues (highly positive in renal tubular cells), according to HPA044540 staining, but weakly positive in most tumor tissues. Acetyl-coenzyme A carboxylase α (ACACA) was strongly positive in most normal renal tissues after staining with the HPA063018 antibody (highly positive in renal tubular cells) but moderately positive in most tumor tissues. Ribosomal protein S6 kinase B1 (RPS6KB1) was weakly or moderately positive in normal renal tissues according to CAB018346 staining but moderately or strongly positive in tumor tissues. Ribonucleotide reductase adjust the M2 (RRM2) was not detected in normal or tumor tissues by HPA056994 antibody staining, but survival analysis of this gene from the HPA database indicated that the 5-year survival rate of patients with high RRM2 expression (39%) was significantly lower than that of patients with low RRM2 expression (77%). Proliferation and apoptosis adaptor protein 15 (PEA15) expression was weakly positive in normal renal tissues but moderately or strongly positive in tumor tissues according to HPA070820 staining. However, a survival analysis from the HPA database showed that the 5-year survival rate of patients with high RRM2 expression was significantly longer than that of patients with low RRM2 expression (P = 0.013). MAPK3 was weakly positive in most normal renal tissues according to CAB002683 staining but moderately positive in most tumor tissues. UGT1A6 was strongly positive in normal renal tissues (renal tubular cells were highly stained) but weakly or moderately positive in tumor tissues after CAB009819 staining. BRAF was strongly positive in most normal renal tissues according to CAB004552 staining (renal tubular cells were highly stained) and weakly or moderately positive in most tumor tissues. EIF4EBP1 was moderately positive in most normal renal tissues according to CAB005032 staining but strongly positive in most tumor tissues. VCL was moderately positive in normal renal tissues according to HOA002131 staining (both glomerular and tubular cells were moderately positive) but weakly positive in tumor tissues. The IGFBP2 survival analysis in the HPA database revealed that the 5-year survival rate of patients in the high-expression group (55%) was significantly lower than those in the low-expression group (72%) (**Fig. 11**).

**Figure 12:**
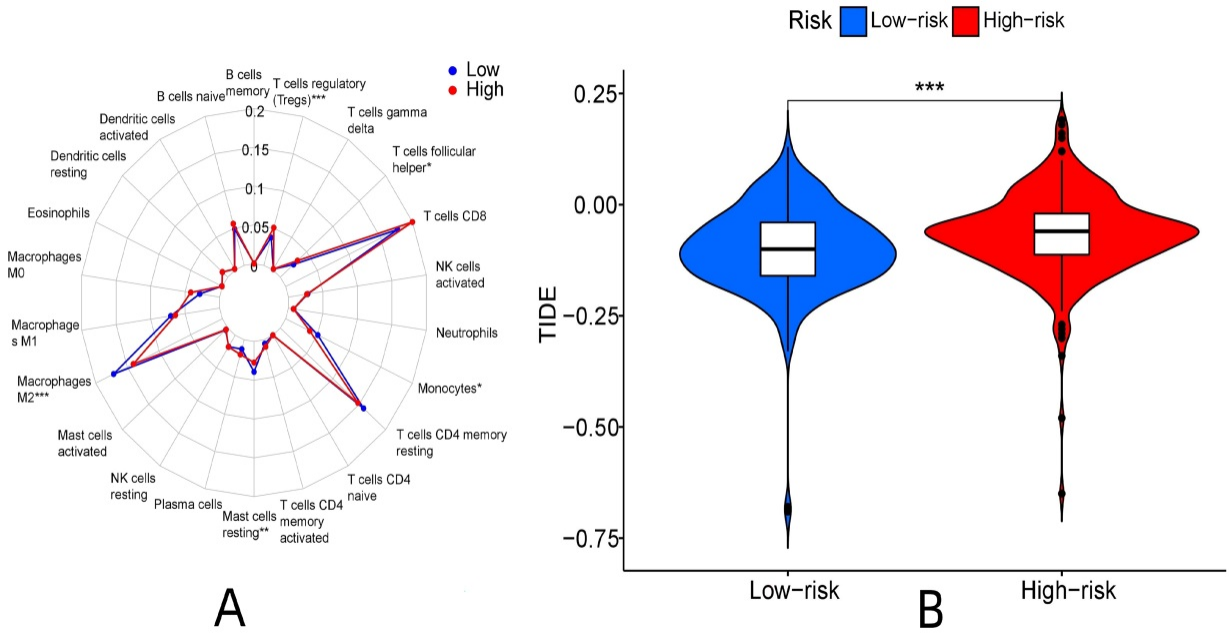
Immune-related function analysis of risk proteins. (A: Analysis of immune cell differences, B: Analysis of TIDE immunotherapy)

There were significant differences in the content of T cells regulatory (P < 0.001), monocytes (P < 0.05), macrophages M2 (P < 0.001), T cells follicular helper (P < 0.05) and mast cells resting (P < 0.01) between the high and low expression groups. Among them, the content of regulatory T cells and follicular helper T cells was higher in the high-expression group than in the low-expression group. Monocytes, resting mast cells, and M2 macrophages were higher in the low-expression group than in the high-expression group (**Fig. 12A**).

Immunotherapy differed between the high and low-risk groups (P < 0.001). The TIDE score of the high-risk group was higher, and the immunotherapeutic effect was worse than that of the low-risk group considering the greater potential of immune escape in the high-risk group (**Fig. 12B**).

### Survival and clinical correlation analyses of the sample types

**Figure 13:**
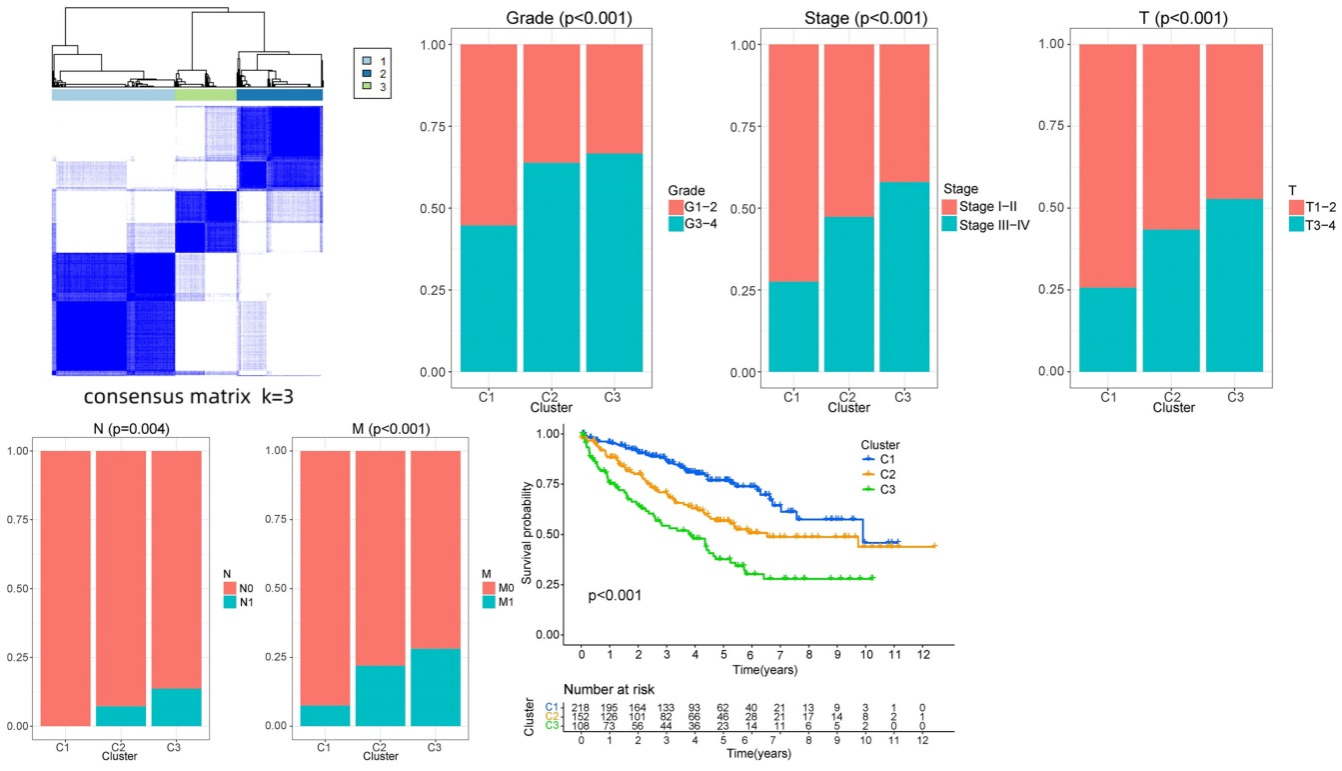
Survival and clinical correlation analyses of the sample types.

The PAM algorithm was used for unsupervised clustering of the expression levels of the risk proteins. The matrix segmentation effect was good when K = 3, noise interference was low, and the data were divided into three subtypes. The correlation analysis of each subtype and the clinical data (age, gender, grade, and T, N and M stage) showed differences in the clinical characteristics among the three subtypes in grade and T, N and M stage, among which cluster3 subtype showed a high proportion in grade, stage and TNM stage. This result indicates that cluster3 subtype tumors have stronger invasive and proliferative abilities. A significant difference in survival time was detected between the subtypes. The survival time of the cluster3 subtype was shorter than that of the cluster2 subtype, and the survival time of the cluster2 subtype was shorter than that of the cluster1 subtype (P < 0.001). With the progression of the tumor, the survival time of the patients was shorter, and the prognosis was worse, indicating that the classification results were consistent with the clinical evidence and the classification results were closely related to the prognosis of the patients (**Fig. 13**).

### Screening of potential drugs for renal clear cell carcinoma

**Figure 14:**
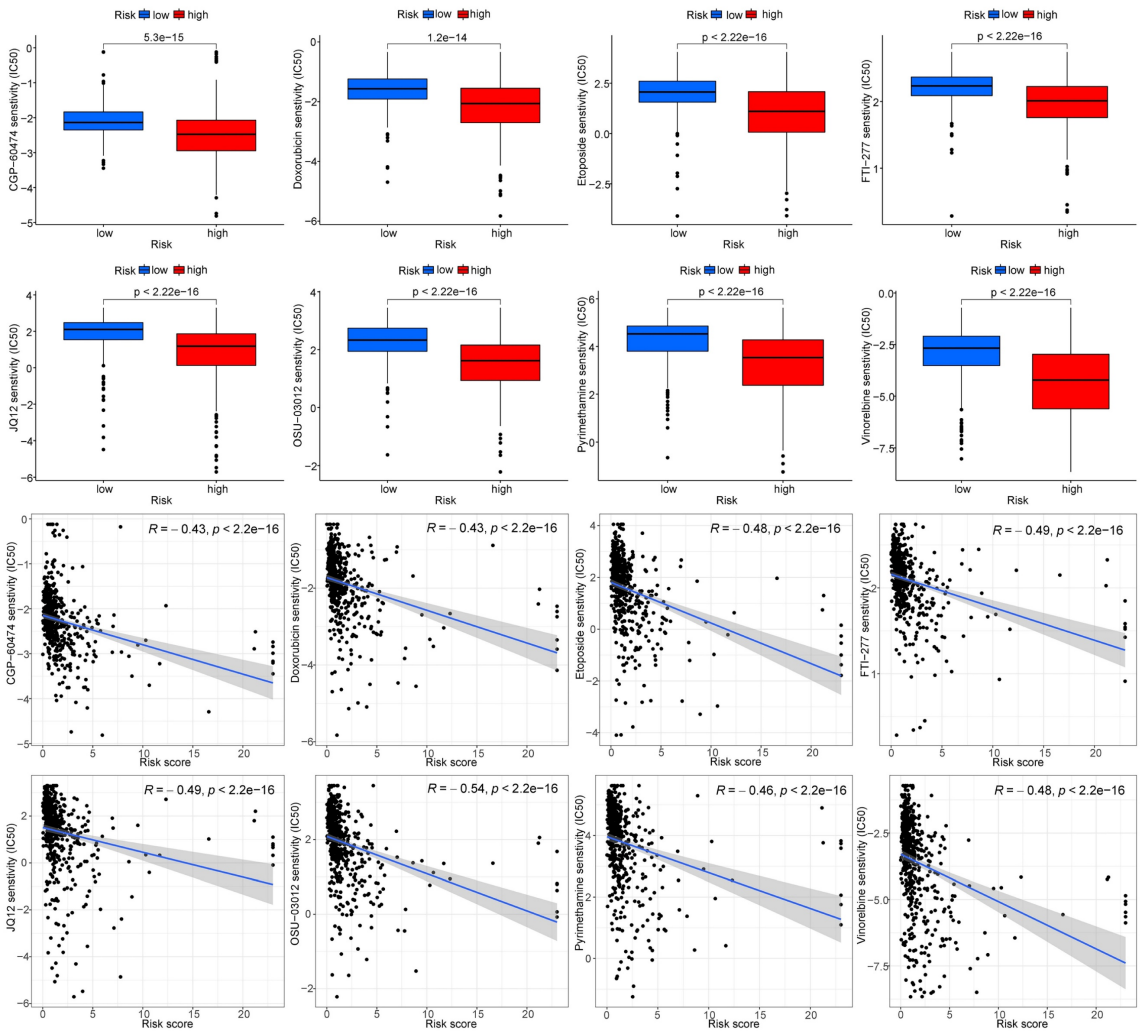
Screening of potential drugs for ccRCC (partial examples)

The gene expression and risk protein data were used to screen potential drugs, and the screening condition was P < 0.001. According to the IC_50_ value, CGP-60474, vinorelbine, doxorubicin, etoposide, FTI-277, JQ12, OSU-03012, pyrimethamine, and other drugs had significantly different effects in the high and low-risk groups, as the high-risk group was more sensitive (**Fig. 14**).

## Discussion

Renal clear cell carcinoma is one of the most common cancers of the urinary system, and its diagnosis and treatment have a definite curative effect in the clinical work. However, recurrence and progression may occur despite surgical treatment, so exploring new ccRCC biomarkers and screening high-risk groups, early for individualized treatment of this class of people, and screen potential drugs 12. To improve the survival rate of renal clear cell carcinoma is necessary. Scholars have identified kidney cancer biomarkers, such as nicotinamide N-methyl transferase, serum amyloid protein, thymidine phosphorylase, and other biomarkers that can be used to diagnose renal cancer 13-16. Biomarkers, such as the S100 family proteins and heat shock proteins, were reported to predict the development, staging, treatment, and prognosis of renal cell carcinoma 17, 18. However, few studies have developed ccRCC-related protein prognostic models.

Therefore, a ccRCC-related protein prognostic model was established based on the TCGA database to predict the prognosis and identify new biomarkers for individualized treatment of high-risk populations.

In this study, 13 proteins with independent prognostic significance were screened by univariate Cox regression, Lasso regression, and multivariate Cox regression analyses to construct the prognostic model. The protein encoded by MITF is a transcription factor that contains basic helix-loop-helix and leucine zipper structural features involved in the lineage-specific regulation of melanocytes, osteoclasts, and mast cells. Recent studies have shown that MITF promotes cell growth, migration, and invasion of ccRCC by activating the RhoA/YAP signaling pathway 19. PTPN11 encodes a protein that is a member of the protein tyrosine phosphatase (PTP) family. PTPs are signaling molecules that regulate various cellular processes, including cell growth, differentiation, the mitotic cycle, and oncogenic transformation. Studies have reported that a mutation in this gene is the cause of Noonan syndrome and acute myeloid leukemia 20, 21. In addition, PTPN11 is hypomethylated in patients with gastric cancer, and PTPN11 hypomethylation may lead to the upregulation of PTPN11 transcripts. The correlation between PTPN11 hypomethylation and the incidence of gastric cancer may be specific to male patients, alcoholic patients, patients with poorly differentiated tumors, and patients with TNM stage III+IV. PTPN11 hypomethylation is a biomarker for recurrence in gastric cancer patients aged ≤ 60 years 22. IGFBP2 is a protein-coding gene that promotes tumor development by inducing alternative polarization of macrophages through the STAT3 pathway in pancreatic ductal adenocarcinoma 23. It also upregulates ZEB through the NF-κB signaling pathway to promote the progression of hepatocellular carcinoma 24. The protein encoded by PRKAA1 (AMP-activated catalytic subunit α1) and PRKAA2 belong to the Ser/Thr protein kinase family and plays a key role in regulating cellular energy metabolism through phosphorylation. Studies have shown that PRKAA1 increases proliferation and inhibits apoptosis of gastric cancer cells by activating the JNK1 and Akt pathways 25. In addition, cyclic CPM promotes chemoresistance in gastric cancer by activating PRKAA2-mediated autophagy 26. ACACA is a protein-coding gene expressed at higher levels in advanced prostate cancer patients than in lower-grade patients. After ACACA knockdown, the proliferation ability of tumor cells decreases, and the downregulation of ACACA prevents the malignant progression of prostate cancer by inhibiting mitochondrial potential 27. BCL2-related protein A1 (BCL2A1) is a member of the BCL-2 protein family, and its related pathways include apoptosis, autophagy, and ALK signaling in cancer.

Studies have shown that BCL2A1 expression is closely related to the occurrence and development of cancers, such as colon cancer, ovarian cancer, and breast cancer 28, 29. High expression of RPS6KB1 in tumor tissues indicates a poor prognosis with poor survival in esophageal cancer patients 30. RRM2 has been reported in many types of cancer and is associated with the development of tumors. A study showed that RRM2 maintains glutathione synthesis in liver cancer cells and plays a role in the resistance to iron die, and through the stable ANXA1 and activation of AKT pathway to regulate kidney to chougny sensitivity for blocking and PD-1^31^, ^32^. PEA15 is a 15 kDa multifunctional phosphoprotein involved in various biological processes, such as the proliferation and apoptosis of cancer cells. Studies have shown that microrNA212-regulated PEA15 promotes the progression of ovarian cancer by inhibiting cell apoptosis 33. The proteins encoded by MAPK1 and MAPK3 are members of the MAP kinase family, and serine/threonine kinases are important components of the MAP kinase signal transduction pathway. MAPK1/ERK2 and MAPK3/ERK1 are two MAPKs that play important roles in the MAPK/ERK cascade and are also involved in the signaling cascade initiated by activated KIT and KITLG/SCF. Phosphorylation of ULK1 by MAPK1/ERK2-MAPK3/ERK1 kinases triggers an interaction with BTRC and subsequent K48-linked ubiquitination and proteasomal degradation, while accumulation of damaged, reactive oxygen species-producing mitochondria leads to activation of the NLRP3 inflammasome. Thus, abnormal soluble cytokine secretion is induced, which, in turn, promotes the differentiation and maturation of osteoclasts, eventually leading to bone metastasis 34. The UGT1A gene family plays important roles in pharmacology and toxicology, leading to differences in drug disposition. Some studies have reported that the differentially expressed UGT1A gene family functions in pancreatic cancer tissues are mainly related to the glucuronylation pathway, cytokine-cytokine receptor interactions, and the ILK signaling pathway. The UGT1A1/3/8/9/10 expression level is positively correlated with the activity of tumor-infiltrating immune cells, particularly B cells. UGT1A6/9 expression is negatively correlated with the level of macrophage infiltration 35. BRAF belongs to the family of RAF serine/threonine protein kinases, and proteins that regulate the MAP kinase/ERK signaling pathway play a role in affecting cell division, differentiation, secretion, and gene mutations. The most common mutation is the V600E mutation, which is most often found in melanoma and a variety of other cancers, including brain tumors, colorectal cancer, and other tumor diseases 36-38. VCL is a cytoskeletal protein associated with cell-cell and cell-matrix junctions. It is thought to be one of several interacting proteins involved in anchoring F-actin to the membrane. It has been proposed that VCL-ALK RCC developed in a 14-year-old girl with the sickle cell trait in ALK-rearranged renal cell carcinoma. Moreover, VCL-ALK RCCS differs from non-VCL-ALK RCCS in that solid structures, and cytoplasmic vacuoles are significantly more frequent in VCL-ALK RCCS than in non-VCL-ALK RCCS 39. Therefore, the model proteins were closely related to the tumor, and survival analysis was carried out on the TCGA database. The ccRCC samples showed that the OS and PFS of the low-risk group were superior to those in the high-risk group, as seen from the survival state and the risk score chart. The number of deaths increased with the risk value, and the risk score was higher. According to the ROC curve and calibration chart, the model had high sensitivity and accuracy for predicting the prognosis of ccRCC, which will provide a potential direction for clinical research.

The GO enrichment analysis revealed that most pathways in the high-risk group were related to immunity, whereas the KEGG enrichment analysis showed that risk proteins in the high-risk group were mainly enriched in cytokine-cytokine receptor interactions, hematopoietic cell lineage, the nod-like receptor signaling pathway, the p53 signaling pathway, primary immunodeficiency, and other related pathways. The model protein was significantly associated with various immune cells, such as regulatory T cells, T cell follicular helper monocytes, resting mast cells, and M2 macrophages. These results show a potential correlation between the model proteins and immune infiltration, and the expression of the model proteins was significantly different between renal tumor tissues and normal tissues according to the immunohistochemical analysis. The TIDE score indicated that the high-risk group had greater potential for immune escape and a poorer immunotherapeutic effect than the low-risk group. Three ccRCC subtypes were identified according to the expression levels of the risk proteins, and the subtype classification was significantly correlated with the clinical prognosis of the patients.

Most of the proteins included in the prognostic risk model were related to the proliferation or death of tumor cells, and some proteins played important roles in the pathogenesis of renal cell carcinoma. This is consistent with the fact that this study is based on ccRCC tumor cell-related risk proteins, which may provide targets and molecular markers for ccRCC-targeted precision therapy in the future. Although the prognostic model must be verified in clinical trials, the prognostic model based on ccRCC-related risk proteins predicted the survival of ccRCC patients more sensitively and accurately than traditional pathological staging. The high-risk ccRCC population can be screened more accurately using this model, and the sensitivity of anti-tumor drugs can be screened by the IC_50_ value to provide an important reference for individualized treatment, and to provide a research direction and theoretical basis for subsequent clinical and experimental research.

Some of the previous related studies mainly focused on identifying one single gene as a therapeutic target in ccRCC. For instance, Lin et al. described the potential significance of NUDT1 as a prognostic biomarker and therapeutic target in ccRCC^40^ and Miao et al. reported that HSD11B2 could serve as a potential biomarker and therapeutic target for ccRCC metastasis^41^. Comparatively, for studies that reported on a panel of genes/proteins, most were limited in terms of either reporting only on a few genes/proteins, assessed only one type of survival (i.e., overall survival only), focused on a singles aspect of ccRCC (i.e., metabolism, ferroptosis, cuproptosis, etc.), or did not thoroughly established potentially significant treatments that could be used to treat the different risk groups of patients their model could stratify. For instance, Tang et al. established a prognostic model on 9 autophagy-related long non-coding RNA^42^, Xu et al. constructed a panel of 10 cuproptosis-associated lncRNAs^43^, Ming et al. designed a panel comprising 12 N7-Methylguanosine (m7G)-related long non-coding RNAs (lncRNAs)^44^, Sun et al. constructed a ferroptosis-related prognostic signature based on 19 ferroptosis-related genes^45^, Liu et al. identified a 13-gene risk signature related to ccRCC patients' metabolism^46^, Zhao et al. described their finding on 3 metabolic genes that were used to build a risk score model^47^, and Peng et al. proposed a 3-gene methylation signature that could be used as a risk stratification tool to predict patient's outcomes and treatment response^48^. Compared these previous literature, to our existing knowledge, this is the first study to thoroughly investigate a panel of 13 proteins in ccRCC based on which we established an algorithm able to differentiate high-risk from low-risk patients, predict their 1-, 3- and 5-year OS, perform immune-related functional analysis to investigate the potential effect of immunotherapeutics in these 2 groups of patients and screen for potential drugs that could have be effective in the high and low-risk groups.

Despite the interesting findings reported in this study, there were some limitations that should be clarified. First, this study was based on bioinformatics analysis retrieved from the online TCGA database, and despite validating the results in a test set, the results should be externally validated. Second, in vivo and in vitro experiments should be performed to validate the actual significance of these potentially promising proteins reported in this study. Third, the survival analysis of this study should be validated in immunohistochemistry studies from clinical ccRCC patient tissues.

## Conclusion

In conclusion, we report the potential clinical usefulness of 13 ccRCC-related proteins (MITF, SHP2_pY542, IGFBP2, AMPKALPHA_pT172, ACC1, P70S6K_pT389, RRM2, PEA15, MAPK_pT202Y204, UGT1A, BRAF_pS445, 4EBP1_pT37T46 and Vinculin) that could be used as a guidance to classify patients into high- and low-risk groups, predict their clinical outcomes and strategize individualized treatments. Although further validations are required to confirm the clinical impact of these proteins and our findings, this study provides a referential basis for improving the outcomes of ccRCC patients.

## Supplementary Material

Supplementary tables.

## Figures and Tables

**Table 1:** Multivariate Cox analysis of proteins associated with ccRCC and the standard names of the proteins

ID of genes from the TCGA	Coefficient	Standard names
**MITF**	1.157314965	MITF
**SHP2_pY542**	0.650254983	PTPN11
**IGFBP2**	0.377159977	IGFBP2
**AMPKALPHA_pT172**	-0.445732648	PRKAA1/PRKAA2
**ACC1**	0.714167488	ACACA
**P70S6K_pT389**	-1.112639445	RPS6KB1
**RRM2**	1.33597513	RRM2
**PEA15**	1.407305683	PEA15
**MAPK_pT202Y204**	-0.549016199	MAPK1/MAPK3
**UGT1A**	-0.314557043	UGT1A
**BRAF_pS445**	-1.450773045	BRAF
**4EBP1_pT37T46`**	0.290217234	EIF4EBP1
**Vinculin**	-0.939738176	VCL

**Table 2:** Clinical characteristics of the study groups.

Variables	Total	Test group	Train group	P-value
**Age**				0.4402
**≤65**	315(65.9%)	162(67.78%)	153(64.02%)	
**>65**	163(34.1%)	77(32.22%)	86(35.98%)	
**Gender**				0.3845
Female	162(33.89%)	86(35.98%)	76(31.8%)	
Male	316(66.11%)	153(64.02%)	163(68.2%)	
**Grade**				0.3845
**G1-2**	162(33.89%)	86(35.98%)	76(31.8%)	
**G3-4**	316(66.11%)	153(64.02%)	163(68.2%)	
**T stage**				0.5707
**T1-2**	299(62.55%)	153(64.02%)	146(61.09%)	
**T3-4**	179(37.45%)	86(35.98%)	93(38.91%)	
**N stage**				0.2933
**N0**	214(44.77%)	109(45.61%)	105(43.93%)	
**N1**	15(3.14%)	5(2.09%)	10(4.18%)	
**Unknown**	249(52.09%)	125(52.3%)	124(51.88%)	
**M stage**				0.3461
M0	381(79.71%)	196(82.01%)	185(77.41%)	
M1	76(15.9%)	34(14.23%)	42(17.57%)	
Unknown	21(4.39%)	9(3.77%)	12(5.02%)	
**TNM stage**				0.3891
**I-II**	282(59%)	147(61.51%)	135(56.49%)	
**III-IV**	193(40.38%)	92(38.49%)	101(42.26%)	
**Unknown**	3(0.63%)	0(0%)	3(1.26%)	

## Abbreviations

ccRCC: renal clear cell carcinoma
RCC: renal cell carcinoma
TCGA: the cancer genome atlas
OS: overall survival
PFS: progression-free survival
ROC: receiver operating characteristic
AUC: area under ROC curve
PCA: principal component analysis
GO: gene ontology
KEGG: kyoto encyclopedia of genes and genomes
MITF: melanocyte inducing transcription factor
PTPN11: protein tyrosine phosphatase non-receptor type 11
IGFBP2: insulin like growth factor binding protein 2
PRKAA1: protein kinase AMP-activated catalytic subunit alpha 1
PRKAA2: protein kinase AMP-activated catalytic subunit alpha 2
ACACA: acetyl-CoA carboxylase alpha
RPS6KB1: ribosomal protein S6 kinase B1
RRM2: ribonucleotide reductase regulatory subunit M2
PEA15: proliferation and apoptosis adaptor protein 15
MAPK1: mitogen-activated protein kinase 1
MAPK2: mitogen-activated protein kinase 2
UGT1A: UDP glucuronosyltransferase family 1 member A complex locus
BRAF: B-Raf proto-oncogene, serine/threonine kinase
EIF4EBP1: eukaryotic translation initiation factor 4E binding protein 1
VCL: vinculin
SHP2_pY542: Src homology-2-containing protein tyrosine phosphatase 2 (pY542)
AMPKALPHA_pT172: 5'-AMP-activated protein kinase α (pT172)
ACC1: acetyl-CoA carboxylase 1
P70S6K: protein 70 S6 kinase
4EBP1: 4E-binding protein 1

## References

[1] Menko FH, Maher ER (2016). Diagnosis and Management of Hereditary Renal Cell Cancer. Recent results in cancer research Fortschritte der Krebsforschung Progres dans les recherches sur le cancer.

[2] Pfaffenroth EC, Linehan WM (2008). Genetic basis for kidney cancer: opportunity for disease-specific approaches to therapy. Expert opinion on biological therapy.

[3] Coleman JA (2008). Familial and hereditary renal cancer syndromes. The Urologic clinics of North America.

[4] Siegel RL, Miller KD, Fuchs HE, Jemal A (2021). Cancer Statistics, 2021. CA: a cancer journal for clinicians.

[5] Patel HD, Gorin MA, Gupta N, Kates M, Johnson MH, Pierorazio PM (2016). Mortality trends and the impact of lymphadenectomy on survival for renal cell carcinoma patients with distant metastasis. Canadian Urological Association journal = Journal de l'Association des urologues du Canada.

[6] Lipworth L, Tarone RE, McLaughlin JK (2011). Renal cell cancer among African Americans: an epidemiologic review. BMC cancer.

[7] Zhang GM, Zhu Y, Gu WJ, Zhang HL, Shi GH, Ye DW (2016). Pretreatment neutrophil-to-lymphocyte ratio predicts prognosis in patients with metastatic renal cell carcinoma receiving targeted therapy. International journal of clinical oncology.

[8] Li CJ, Li MC (2017). Research progress of molecular targeted therapy and combination treatment against renal cell carcinoma. Chinese Journal of Hospital Pharmacy.

[9] Hsieh JJ, Purdue MP, Signoretti S, Swanton C, Albiges L, Schmidinger M (2017). Renal cell carcinoma. Nature reviews Disease primers.

[10] Kumar R, Kapoor A (2017). Current management of metastatic renal cell carcinoma: evolving new therapies. Current opinion in supportive and palliative care.

[11] Bedke J, Gauler T, Grunwald V, Hegele A, Herrmann E, Hinz S (2017). Systemic therapy in metastatic renal cell carcinoma. World journal of urology.

[12] Huang J, Wang Y, Zhang H, Hu X, Wang P, Cai W (2021). Clinical outcomes of second-line treatment following first-line VEGFR-TKI failure in patients with metastatic renal cell carcinoma: a comparison of axitinib alone and axitinib plus anti-PD-1 antibody. Cancer communications.

[13] Kim DS, Choi YP, Kang S, Gao MQ, Kim B, Park HR (2010). Panel of candidate biomarkers for renal cell carcinoma. Journal of proteome research.

[14] Wood SL, Rogers M, Cairns DA, Paul A, Thompson D, Vasudev NS (2010). Association of serum amyloid A protein and peptide fragments with prognosis in renal cancer. British journal of cancer.

[15] Li HC, Lei J, Liu QW, Tan J (2015). Expression of Thymidine Phosphorylase in Tumor Cells and Its Correlation with Curative Efficacy of Capecitabine. Practical Clinical Medicine.

[16] Wang X, Xu H, Guo M, Shen Y, Li P, Wang Z (2021). The use of an oxidative stress scoring system in prognostic prediction for kidney renal clear cell carcinoma. Cancer communications.

[17] Gabril M, Girgis H, Scorilas A, Rotondo F, Wala S, Bjarnason GA (2016). S100A11 is a potential prognostic marker for clear cell renal cell carcinoma. Clinical & experimental metastasis.

[18] Fu Y, Xu X, Huang D, Cui D, Liu L, Liu J (2017). Plasma Heat Shock Protein 90alpha as a Biomarker for the Diagnosis of Liver Cancer: An Official, Large-scale, and Multicenter Clinical Trial. EBioMedicine.

[19] Kim N, Kim S, Lee MW, Jeon HJ, Ryu H, Kim JM (2021). MITF Promotes Cell Growth, Migration and Invasion in Clear Cell Renal Cell Carcinoma by Activating the RhoA/YAP Signal Pathway. Cancers.

[20] Stasik S, Eckardt JN, Kramer M, Rollig C, Kramer A, Scholl S (2021). Impact of PTPN11 mutations on clinical outcome analyzed in 1529 patients with acute myeloid leukemia. Blood advances.

[21] Athota JP, Bhat M, Nampoothiri S, Gowrishankar K, Narayanachar SG, Puttamallesh V (2020). Molecular and clinical studies in 107 Noonan syndrome affected individuals with PTPN11 mutations. BMC medical genetics.

[22] Xu L, Zhou C, Pan R, Tang J, Wang J, Li B (2020). PTPN11 hypomethylation is associated with gastric cancer progression. Oncology letters.

[23] Sun L, Zhang X, Song Q, Liu L, Forbes E, Tian W (2021). IGFBP2 promotes tumor progression by inducing alternative polarization of macrophages in pancreatic ductal adenocarcinoma through the STAT3 pathway. Cancer letters.

[24] Guo Q, Yu DY, Yang ZF, Liu DY, Cao HQ, Liao XW (2020). IGFBP2 upregulates ZEB1 expression and promotes hepatocellular carcinoma progression through NF-kappaB signaling pathway. Digestive and liver disease: official journal of the Italian Society of Gastroenterology and the Italian Association for the Study of the Liver.

[25] Zhang Y, Zhou X, Cheng L, Wang X, Zhang Q, Zhang Y (2020). PRKAA1 Promotes Proliferation and Inhibits Apoptosis of Gastric Cancer Cells Through Activating JNK1 and Akt Pathways. Oncology research.

[26] Fang L, Lv J, Xuan Z, Li B, Li Z, He Z (2022). Circular CPM promotes chemoresistance of gastric cancer via activating PRKAA2-mediated autophagy. Clinical and translational medicine.

[27] Zhang H, Liu S, Cai Z, Dong W, Ye J, Cai Z (2021). Down-regulation of ACACA suppresses the malignant progression of Prostate Cancer through inhibiting mitochondrial potential. Journal of Cancer.

[28] Yue T, Liu X, Zuo S, Zhu J, Li J, Liu Y (2021). BCL2A1 and CCL18 Are Predictive Biomarkers of Cisplatin Chemotherapy and Immunotherapy in Colon Cancer Patients. Frontiers in cell and developmental biology.

[29] Liang R, Yung MMH, He F, Jiao P, Chan KKL, Ngan HYS (2021). The Stress-Inducible BCL2A1 Is Required for Ovarian Cancer Metastatic Progression in the Peritoneal Microenvironment. Cancers.

[30] Wang L, Ji XB, Wang LH, Xia ZK, Xie YX, Liu WJ (2021). MiRNA-30e downregulation increases cancer cell proliferation, invasion and tumor growth through targeting RPS6KB1. Aging.

[31] Yang Y, Lin J, Guo S, Xue X, Wang Y, Qiu S (2020). RRM2 protects against ferroptosis and is a tumor biomarker for liver cancer. Cancer cell international.

[32] Xiong W, Zhang B, Yu H, Zhu L, Yi L, Jin X (2021). RRM2 Regulates Sensitivity to Sunitinib and PD-1 Blockade in Renal Cancer by Stabilizing ANXA1 and Activating the AKT Pathway. Advanced science.

[33] Luo Y, Fang C, Jin L, Ding H, Lyu Y, Ni G (2020). The microRNA212 regulated PEA15 promotes ovarian cancer progression by inhibiting of apoptosis. Journal of Cancer.

[34] Deng R, Zhang HL, Huang JH, Cai RZ, Wang Y, Chen YH (2021). MAPK1/3 kinase-dependent ULK1 degradation attenuates mitophagy and promotes breast cancer bone metastasis. Autophagy.

[35] Feng L, Wang Y, Qin J, Fu Y, Guo Z, Zhang J (2021). UGT1A Gene Family Members Serve as Potential Targets and Prognostic Biomarkers for Pancreatic Cancer. BioMed research international.

[36] Ritterhouse LL, Barletta JA (2015). BRAF V600E mutation-specific antibody: A review. Seminars in diagnostic pathology.

[37] Schreck KC, Grossman SA, Pratilas CA (2019). BRAF Mutations and the Utility of RAF and MEK Inhibitors in Primary Brain Tumors. Cancers.

[38] Grassi E, Corbelli J, Papiani G, Barbera MA, Gazzaneo F, Tamberi S (2021). Current Therapeutic Strategies in BRAF-Mutant Metastatic Colorectal Cancer. Frontiers in oncology.

[39] Wangsiricharoen S, Zhong M, Ranganathan S, Matoso A, Argani P (2021). ALK-rearranged Renal Cell Carcinoma (RCC): A Report of 2 Cases and Review of the Literature Emphasizing the Distinction Between VCL-ALK and Non-VCL-ALK RCC. International journal of surgical pathology.

[40] Lin Y, Zhang F, Jin Y, Zhong Q, Tan W, Liu J (2022). NUDT1 Could Be a Prognostic Biomarker and Correlated with Immune Infiltration in Clear Cell Renal Cell Carcinoma. Applied bionics and biomechanics.

[41] Miao S, Song J, Liu Q, Lai J, Wang H, Ran L (2022). Integrated bioinformatics analysis to identify the key gene associated with metastatic clear cell renal cell carcinoma. Medical oncology.

[42] Tang F, Tang Z, Lu Z, Cai Y, Lai Y, Mai Y (2022). A novel autophagy-related long non-coding RNAs prognostic risk score for clear cell renal cell carcinoma. BMC urology.

[43] Xu S, Liu D, Chang T, Wen X, Ma S, Sun G (2022). Cuproptosis-Associated lncRNA Establishes New Prognostic Profile and Predicts Immunotherapy Response in Clear Cell Renal Cell Carcinoma. Frontiers in genetics.

[44] Ming J, Wang C (2022). N7-Methylguanosine-Related lncRNAs: Integrated Analysis Associated With Prognosis and Progression in Clear Cell Renal Cell Carcinoma. Frontiers in genetics.

[45] Sun Z, Li T, Xiao C, Zou S, Zhang M, Zhang Q (2022). Prediction of overall survival based upon a new ferroptosis-related gene signature in patients with clear cell renal cell carcinoma. World journal of surgical oncology.

[46] Liu S, Zhou H, Wang G, Lian X (2021). Comprehensive Transcriptomic Analysis of Critical RNA Regulation Associated With Metabolism and Prognosis in Clear Cell Renal Carcinoma. Frontiers in cell and developmental biology.

[47] Zhao Y, Tao Z, Chen X (2020). A Three-Metabolic-Genes Risk Score Model Predicts Overall Survival in Clear Cell Renal Cell Carcinoma Patients. Frontiers in oncology.

[48] Peng Q, Zhou Y, Jin L, Cao C, Gao C, Zhou J (2020). Development and validation of an integrative methylation signature and nomogram for predicting survival in clear cell renal cell carcinoma. Translational andrology and urology.

