# Supplementary material for: Construction and Validation of Protein Expression-related Prognostic Models in Clear Cell Renal Cell Carcinoma
Source: J Cancer. 2023 Mar 27;14(5):793–808. doi: 10.7150/jca.81915 (PMC10088890; doi:10.7150/jca.81915)
Supplement: Supplementary file 1 — Supplementary tables. [file jcav14p0793s1.pdf]

Supplementary Table S1. Univariate cox analysis

id	HR	HR.95L	HR.95H	P-value
**P27_pT198**	7.889743295	2.420179068	25.72043123	0.000612826
**CRAF_pS338**	0.05518242	0.005912442	0.515032496	0.011015547
**IGFBP3**	1.815465695	1.155055151	2.853470405	0.009744923
**BAX**	2.061781826	1.321198981	3.217489839	0.001439147
**EPPK1**	0.747476402	0.64062917	0.872144132	0.000217145
**P90RSK**	0.207050709	0.098379766	0.435760294	3.36E-05
**PLC-gamma2_pY759**	2.680225292	1.246827779	5.761507512	0.011571896
**HER3_pY1289**	0.214822077	0.081740962	0.564570365	0.001811042
**MIG6**	0.326115551	0.193045152	0.550914391	2.81E-05
**P53**	1.672970839	1.017636266	2.750325948	0.042469336
**CD31**	0.238313828	0.097269559	0.583877226	0.001707885
**ASNS**	1.969869278	1.374290785	2.823554533	0.000223562
**Hexokinase-I**	2.266132488	1.018641981	5.041375233	0.04493762
**BCLXL**	2.718840765	1.603001951	4.611407427	0.000206838
**MITF**	2.439451638	1.094215765	5.438529116	0.029251877
**CJUN_pS73**	0.386834481	0.163947152	0.912738729	0.030126209
**CMYC**	1.942056736	1.267315382	2.976042443	0.002305634
**YAP_pS127**	1.893182851	1.29452075	2.768701318	0.000998373
**53BP1**	1.861904949	1.093332961	3.170754164	0.02210988
**SHP2_pY542**	0.236592718	0.150992311	0.370721621	3.17E-10
**EGFR**	1.744814255	1.019521769	2.986083156	0.042307014
**S100A4**	2.043719952	1.227867853	3.40166186	0.005965866
**PAX6**	2.259192422	1.022657816	4.990868226	0.043866169
**ATM_pS1981**	1.342429168	1.011532676	1.781569806	0.04141267
**SHC_pY317**	0.18869139	0.093463221	0.380946005	3.28E-06
**ACC_pS79**	1.986050933	1.28063677	3.080028936	0.002177827
**DRP1**	2.513363886	1.584251125	3.987371649	9.08E-05
**CYCLINB1**	2.009656258	1.543243629	2.617032204	2.22E-07
**ULK1_pS757**	0.117142478	0.037357164	0.367328746	0.000235542
**MMP14**	2.32867129	1.583817538	3.423822408	1.72E-05
**EEF2**	1.671855505	1.22345116	2.284603522	0.001256083
**Cyclin-D3**	3.052567567	1.117732852	8.336668942	0.02947338
**FAK_pY397**	0.255414274	0.150901574	0.432311274	3.71E-07
**CHK2**	2.978733971	1.35245005	6.560579494	0.006740299
**NFKBP65_pS536**	0.651270867	0.48051169	0.882712639	0.005708033
**Lyn**	2.564906154	1.168273703	5.631166365	0.018895266
**EGFR_pY1068**	0.648898411	0.512609321	0.821423121	0.000323977
**VHL**	8.246505436	2.306013442	29.49022355	0.001174158
**IGFBP2**	2.087947267	1.498675828	2.90891713	1.35E-05
**BETACATENIN**	0.490059977	0.367423056	0.653630133	1.21E-06
**SYK**	1.569851825	1.079110291	2.283765406	0.01837052
**PRAS40_pT246**	0.622759996	0.419296318	0.924954491	0.018951818
**CASPASE7CLEAVEDD198**	1.664960651	1.049699554	2.640845141	0.030307516
**CYCLINE1**	2.478031196	1.261846936	4.866389444	0.008403895
**NDRG1_pT346**	0.747363118	0.635565208	0.87882663	0.000427735
**AMPKALPHA_pT172**	0.382891882	0.272297319	0.538404837	3.39E-08
**DDR1_pY513**	0.142573514	0.036926933	0.550471034	0.004712033

**CREB_pS133**	0.534776113	0.313530077	0.912146911	0.02159041
**MIF**	0.403159615	0.192923078	0.842499903	0.01570465
**Cox2**	2.465962002	1.280245469	4.749845826	0.006962719
**1433ZETA**	2.80043871	1.725924321	4.543917062	3.05E-05
**Caspase-8-cleaved**	0.118965896	0.038459978	0.367989928	0.000219779
**Myt1**	9.756928644	1.756207489	54.20638346	0.00922431
**FRS2-alpha_pY196**	0.031089754	0.006449061	0.149878065	1.53E-05
**IGFRb**	0.300060483	0.153579637	0.586251504	0.000427252
**D-a-Tubulin**	5.571378138	2.931293066	10.58927021	1.59E-07
**HER3**	0.395062979	0.259514066	0.601411553	1.48E-05
**XRCC1**	2.465866523	1.22115665	4.97929378	0.011829017
**Cyclophilin-F**	1.420648789	1.091228231	1.849514999	0.009091754
**AKT**	0.316752614	0.156453286	0.641291857	0.001401024
**ACC1**	2.50097418	1.747777797	3.578756899	5.33E-07
**CD26**	0.60421963	0.413962046	0.881919887	0.009022249
**IR-b**	0.517522336	0.370081363	0.723704014	0.000118098
**SMAC**	1.92025721	1.366695973	2.698030744	0.000169595
**BAD_pS112**	2.224520717	1.066914136	4.638135586	0.032946392
**CHK1**	4.443012226	2.044729752	9.654262442	0.000165636
**MEK1**	0.4281262	0.280795055	0.652760938	8.08E-05
**MEK1_pS217S221**	0.294753664	0.146534048	0.592897854	0.000612696
**MSH6**	4.524071453	2.129231336	9.612493563	8.66E-05
**G6PD**	1.606477213	1.148033474	2.247991104	0.00568775
**DNMT1**	5.640140953	2.364250328	13.45508536	9.63E-05
**ANNEXINVII**	0.346637203	0.122436686	0.981383559	0.046004115
**H2AX_pS139**	4.409940172	1.386734685	14.02400368	0.011941596
**AMPKALPHA**	0.591827462	0.360723072	0.970993464	0.037848648
**CMET_pY1235**	3.124532701	1.005535881	9.708956968	0.048894831
**Mnk1**	3.504199794	1.240874854	9.89577326	0.017912861
**4EBP1**	1.981937285	1.25270259	3.135680754	0.003472282
**RSK1**	0.439028089	0.238922378	0.806729219	0.008006166
**GSK3ALPHABETA_pS21S9**	0.596985237	0.411058164	0.867009597	0.006738361
**XPA**	0.261208717	0.12611941	0.540995184	0.00030182
**PLK1**	4.102234541	2.516725782	6.686595872	1.49E-08
**Aurora-A**	6.927785876	2.222321955	21.59642847	0.000848324
**PYGL**	0.757683849	0.607794056	0.944538382	0.01361339
**JNK2**	0.213063133	0.094689354	0.479419241	0.000186436
**HER2_pY1248**	0.409279668	0.197446214	0.848382164	0.016302599
**P70S6K_pT389**	0.255476085	0.109323339	0.597018268	0.001627188
**GSK3_pS9**	0.554594683	0.352547984	0.872435175	0.010761796
**DNA_POLG**	0.46964112	0.230627452	0.956359615	0.037256716
**p44-42-MAPK**	0.410621086	0.197935668	0.851840794	0.016818121
**CHK1_pS296**	3.712051396	1.164362579	11.83422228	0.026608666
**TRIP13**	6.196826612	2.3608159	16.26584269	0.000211727
**CD171**	1.276716321	1.093965621	1.48999615	0.001939111
**b-Actin**	9.128200674	2.45570403	33.93081842	0.000963048
**YB1**	2.272632233	1.340236224	3.85369174	0.002312667
**PERK**	4.739934502	2.188226521	10.26720902	7.96E-05
**RICTOR**	0.743515211	0.554708865	0.996585605	0.0473845

**P38_pT180Y182**	0.706821259	0.516552343	0.967174574	0.030116026
**PAR**	1.59731695	1.182465773	2.157712719	0.002270883
**MMP2**	5.124790556	1.546068789	16.98726372	0.007526932
**MTOR_pS2448**	0.182468225	0.062780626	0.530333246	0.001777511
**MR1**	0.301844164	0.09524389	0.956595744	0.041814212
**1433BETA**	2.640388569	1.256560712	5.548201314	0.010384032
**b-Catenin_pT41_S45**	9.97397992	1.858757117	53.51978187	0.007293316
**c-IAP2**	0.136050448	0.038398185	0.482046861	0.00199784
**SMAD3**	3.00864035	1.522093257	5.947018498	0.001533392
**TFRC**	1.919331576	1.482921746	2.484172689	7.28E-07
**Pyk2_pY402**	0.203441439	0.103370528	0.40038897	4.03E-06
**STATHMIN**	2.263741172	1.033102967	4.960322694	0.041217765
**RAB11**	0.58359991	0.422631079	0.805877447	0.001072688
**CD86**	4.073623481	1.543366727	10.75208372	0.004563725
**RRM2**	19.09876746	4.983768061	73.19018744	1.68E-05
**ERALPHA**	0.505281239	0.258216226	0.988741626	0.046259246
**DM-K9-Histone-H3**	3.443586757	1.064545633	11.1392968	0.0389808
**MRAP**	8.530731411	1.172658044	62.05848226	0.034236471
**Cdc42**	9.138145973	2.363360059	35.33347004	0.001343767
**SRC_pY416**	0.527237583	0.337094489	0.824633678	0.005033823
**HER2**	0.464726365	0.250301555	0.8628416	0.015214197
**SRC_pY527**	0.437466145	0.32936401	0.581048998	1.14E-08
**PEA15**	6.252464419	3.19533075	12.23451166	8.71E-08
**PTEN**	0.325332392	0.194452895	0.544302335	1.90E-05
**Porin**	2.261956169	1.016967385	5.031081413	0.045369195
**Atg4B**	2.497521307	1.35783922	4.593778547	0.003242288
**FOXO3A_pS318S321**	3.952374396	1.384539869	11.28263889	0.010231227
**CD49B**	2.877947821	1.376793983	6.015848243	0.004954883
**TSC1**	0.342179894	0.182852624	0.640335794	0.000796076
**PCADHERIN**	3.891872353	1.399226114	10.82503411	0.009225858
**FASN**	2.383357005	1.531316811	3.709480999	0.000119127
**MAPK_pT202Y204**	0.453005918	0.345701546	0.593617135	9.40E-09
**PDK1_pS241**	0.352538729	0.190120079	0.653710834	0.000935619
**Cdc6**	7.270044779	1.999366417	26.43514997	0.002596549
**GAB2**	0.501692499	0.37646185	0.668581329	2.50E-06
**FIBRONECTIN**	1.576327026	1.111345461	2.235854629	0.010712047
**AR**	0.335516316	0.199220125	0.565059372	4.02E-05
**PAXILLIN**	0.624894582	0.411647916	0.948609779	0.027266081
**UGT1A**	0.556067938	0.41004562	0.754090609	0.000159407
**TUBERIN**	0.399464928	0.246158129	0.648250901	0.000203376
**FTO**	0.56387062	0.322288407	0.98653898	0.044701834
**BRAF_pS445**	0.358820747	0.160370541	0.802842767	0.012617006
**GCLC**	0.286260363	0.179145453	0.457421578	1.69E-07
**RAPTOR**	2.133296167	1.188150004	3.830284492	0.011171394
**CABL**	4.766237829	1.314528016	17.28150543	0.017497085
**TIGAR**	2.455269484	1.34822294	4.471328931	0.003315305
**ERK2**	0.70298968	0.50699293	0.974756175	0.034576391
**Mitofusin-1**	1.76168199	1.118961589	2.773574594	0.014471077
**XIAP**	0.335888037	0.14662464	0.769453031	0.009890074

**CDK1_pT14**	3.315852033	2.245054963	4.897374403	1.70E-09
**FN14**	2.863072213	1.492245631	5.493185793	0.001556391
**VASP**	3.088143805	1.746855014	5.459315217	0.000104944
**Cox-IV**	1.481220812	1.006704997	2.179402208	0.046165207
**PEA15_pS116**	1.386534509	1.01164341	1.900351376	0.042159815
**YB1_pS102**	3.179216575	1.716885007	5.88706756	0.000233785
**ERALPHA_pS118**	2.056427931	1.129954756	3.742535543	0.018281109
**AKT_pS473**	0.453984255	0.31270962	0.65908335	3.30E-05
**Enolase-1**	0.673247147	0.483009888	0.938410855	0.019535616
**4EBP1_pT70**	3.023307895	1.08446503	8.428478906	0.034432088
**EMA**	1.406349421	1.109688588	1.782318677	0.004787602
**IGF1R_pY1135Y1136**	0.050936806	0.013136819	0.19750277	1.66E-05
**Glucocorticoid-Receptor**	0.5517246	0.378213472	0.804836572	0.002022203
**GYS_pS641**	0.752835067	0.591531189	0.958124692	0.021017705
**PCNA**	3.998317566	1.228137807	13.01689702	0.021381076
**GATA6**	0.413962294	0.195386418	0.87705575	0.021311285
**S6**	2.458473752	1.449350888	4.17020698	0.000848534
**RPA32_pS4_S8**	6.947711783	1.034468482	46.66231969	0.046060531
**SRC**	2.134220869	1.105034714	4.121950798	0.023985459
**STAT3_pY705**	0.588015617	0.355998633	0.97124633	0.038087464
**P21**	5.444409696	2.783721051	10.64819225	7.37E-07
**DNA-Ligase-IV**	0.468694319	0.228984217	0.959342819	0.038122367
**CSK**	4.569987709	1.071229025	19.49609949	0.040079881
**4EBP1_pT37T46**	1.635254922	1.153944617	2.317319755	0.005692575
**Wee1_pS642**	21.45318929	3.846634816	119.6472638	0.000471759
**PAI1**	1.600388818	1.358871338	1.884832138	1.76E-08
**SCD1**	0.32817265	0.114146189	0.943503145	0.038649788
**RAD51**	1.702124984	1.138566749	2.544628555	0.009528278
**Vinculin**	0.490129297	0.303844883	0.790622917	0.003467116
**CD44**	2.534502161	1.9629041	3.27254969	9.89E-13
**DVL3**	2.788103518	1.341180647	5.796028479	0.006029713
**CHD1L**	3.583065325	1.024845955	12.52710913	0.045673952
**TUBERIN_pT1462**	0.218859266	0.105121658	0.455656607	4.89E-05

Supplementary Table S2. Correlations between model proteins and other proteins

protein1	protein2	cor	P-value
**4EBP1_pT37T46**	S6_pS235S236	0.564	1.57E-41
**4EBP1_pT37T46**	S6_pS240S244	0.531	3.54E-36
**4EBP1_pT37T46**	4EBP1_pT70	0.403	4.12E-20
**ACC1**	ACC_pS79	0.885	6.62E-160
**ACC1**	PERK	0.548	6.95E-39
**ACC1**	FASN	0.534	1.29E-36
**ACC1**	EEF2	0.477	1.44E-28
**ACC1**	CDK1_pT14	0.476	1.96E-28
**ACC1**	DRP1	0.464	6.23E-27
**ACC1**	CHK2	0.455	8.09E-26
**ACC1**	S6	0.447	6.66E-25
**ACC1**	Cyclophilin-F	0.446	1.02E-24
**ACC1**	XPA	-0.444	1.82E-24
**ACC1**	PLK1	0.439	6.78E-24
**ACC1**	HER2_pY1248	-0.427	1.17E-22
**ACC1**	NOTCH1	-0.416	2.23E-21
**ACC1**	RAB11	-0.408	1.24E-20
**ACC1**	CYCLINB1	0.408	1.25E-20
**ACC1**	Mitofusin-1	0.401	7.43E-20
**AMPKALPHA_pT172**	SRC_pY527	0.513	2.21E-33
**AMPKALPHA_pT172**	BETACATENIN	0.503	5.87E-32
**AMPKALPHA_pT172**	PTEN	0.499	1.67E-31
**AMPKALPHA_pT172**	TUBERIN	0.467	2.82E-27
**AMPKALPHA_pT172**	ATM	0.437	1.03E-23
**AMPKALPHA_pT172**	P38MAPK	0.429	7.18E-23
**AMPKALPHA_pT172**	AMPKALPHA	0.409	9.94E-21
**AMPKALPHA_pT172**	TSC1	0.406	2.04E-20
**AMPKALPHA_pT172**	AKT	0.401	6.26E-20
**BRAF_pS445**	p38-a	0.638	6.42E-56
**BRAF_pS445**	BRAF	0.634	3.69E-55
**BRAF_pS445**	p44-42-MAPK	0.588	7.63E-46
**BRAF_pS445**	P38MAPK	0.562	3.15E-41
**BRAF_pS445**	ERCC5	0.551	2.32E-39
**BRAF_pS445**	RB	-0.547	9.96E-39
**BRAF_pS445**	ARAF	0.526	2.22E-35
**BRAF_pS445**	PKA-a	0.515	1.12E-33
**BRAF_pS445**	ETS1	-0.511	3.18E-33
**BRAF_pS445**	AMPKALPHA	0.511	3.29E-33
**BRAF_pS445**	AceCS1	0.509	6.25E-33
**BRAF_pS445**	GSK-3B	0.494	9.04E-31
**BRAF_pS445**	PAK1	0.494	9.09E-31
**BRAF_pS445**	MERIT40	0.49	2.97E-30
**BRAF_pS445**	RPA32	0.479	9.16E-29
**BRAF_pS445**	GSK3ALPHABETA	0.477	1.39E-28
**BRAF_pS445**	MTOR	0.474	4.26E-28
**BRAF_pS445**	PDK1_pS241	0.472	7.74E-28
**BRAF_pS445**	STATHMIN	-0.47	1.25E-27

**BRAF_pS445**	PAICS	0.46	2.32E-26
**BRAF_pS445**	DJ1	0.458	3.38E-26
**BRAF_pS445**	PAX8	0.457	4.32E-26
**BRAF_pS445**	Akt2	0.457	4.63E-26
**BRAF_pS445**	MR1	-0.455	8.70E-26
**BRAF_pS445**	RSK1	0.454	1.25E-25
**BRAF_pS445**	BID	-0.447	6.91E-25
**BRAF_pS445**	TRAP1	0.439	6.10E-24
**BRAF_pS445**	ALKBH5	0.433	2.95E-23
**BRAF_pS445**	Stat3	0.431	4.95E-23
**BRAF_pS445**	CMYC	-0.43	6.55E-23
**BRAF_pS445**	YTHDF2	0.428	1.12E-22
**BRAF_pS445**	TRIM25	0.427	1.24E-22
**BRAF_pS445**	MRE11	-0.42	6.82E-22
**BRAF_pS445**	CREB_pS133	0.412	5.08E-21
**BRAF_pS445**	Erk5	0.41	9.18E-21
**BRAF_pS445**	Gli3	-0.408	1.44E-20
**BRAF_pS445**	MIF	0.407	1.65E-20
**BRAF_pS445**	RAD51	-0.405	2.73E-20
**BRAF_pS445**	MTOR_pS2448	0.404	3.32E-20
**BRAF_pS445**	MLH1	0.404	3.49E-20
**IGFBP2**	P21	0.435	1.76E-23
**IGFBP2**	CYCLINB1	0.402	5.43E-20
**MAPK_pT202Y204**	SHC_pY317	0.656	3.19E-60
**MAPK_pT202Y204**	SHP2_pY542	0.653	2.02E-59
**MAPK_pT202Y204**	SRC_pY527	0.569	2.54E-42
**MAPK_pT202Y204**	AKT_pS473	0.534	1.15E-36
**MAPK_pT202Y204**	STAT3_pY705	0.521	1.46E-34
**MAPK_pT202Y204**	Pyk2_pY402	0.482	3.43E-29
**MAPK_pT202Y204**	SRC_pY416	0.481	5.15E-29
**MAPK_pT202Y204**	MEK1_pS217S221	0.477	1.72E-28
**MAPK_pT202Y204**	FAK_pY397	0.431	4.98E-23
**MAPK_pT202Y204**	ULK1_pS757	0.42	6.73E-22
**MAPK_pT202Y204**	IGF1R_pY1135Y1136	0.415	2.44E-21
**MAPK_pT202Y204**	CDK1_pT14	-0.406	2.34E-20
**MITF**	SGK3	0.556	4.72E-40
**MITF**	CtIP	0.548	7.83E-39
**MITF**	UVRAG	0.547	1.14E-38
**MITF**	Coup-TFII	0.545	2.66E-38
**MITF**	Creb	0.524	4.88E-35
**MITF**	ATM_pS1981	0.522	9.20E-35
**MITF**	IL-6	0.522	1.07E-34
**MITF**	Elk1_pS383	0.513	1.95E-33
**MITF**	Akt2_pS474	0.503	4.78E-32
**MITF**	XPF	0.495	7.41E-31
**MITF**	EphA2	0.49	2.70E-30
**MITF**	4-Oct	0.485	1.47E-29
**MITF**	Notch1-cleaved	0.485	1.65E-29
**MITF**	PHLPP	0.482	3.16E-29

**MITF**	Aurora-B	0.464	7.52E-27
**MITF**	Sox2	0.46	1.91E-26
**MITF**	PAX6	0.456	6.14E-26
**MITF**	CDT1	0.452	2.01E-25
**MITF**	ZEB1	0.444	1.53E-24
**MITF**	EphA2_pY588	0.414	3.35E-21
**MITF**	MDM2_pS166	0.409	1.02E-20
**MITF**	WIPI1	0.408	1.24E-20
**MITF**	B7-H4	0.401	7.24E-20
**P70S6K_pT389**	CRAF_pS338	0.684	4.22E-67
**P70S6K_pT389**	HER3_pY1289	0.602	1.78E-48
**P70S6K_pT389**	PR	0.587	1.09E-45
**P70S6K_pT389**	CHK2_pT68	0.571	1.19E-42
**P70S6K_pT389**	RICTOR_pT1135	0.569	2.59E-42
**P70S6K_pT389**	RAD51	0.544	3.12E-38
**P70S6K_pT389**	FOXM1	0.516	6.11E-34
**P70S6K_pT389**	STATHMIN	0.515	8.47E-34
**P70S6K_pT389**	GSK3ALPHABETA	-0.509	8.25E-33
**P70S6K_pT389**	IGF1R_pY1135Y1136	0.498	2.46E-31
**P70S6K_pT389**	AKT	-0.494	8.69E-31
**P70S6K_pT389**	IRS1	0.488	5.80E-30
**P70S6K_pT389**	ULK1_pS757	0.486	1.15E-29
**P70S6K_pT389**	JAK2	-0.475	2.49E-28
**P70S6K_pT389**	eIF4E_pS209	0.474	4.33E-28
**P70S6K_pT389**	MTOR	-0.464	6.00E-27
**P70S6K_pT389**	PARG	0.458	3.31E-26
**P70S6K_pT389**	P53	0.456	5.93E-26
**P70S6K_pT389**	BRD4	-0.455	8.64E-26
**P70S6K_pT389**	FOXO3A	0.45	2.93E-25
**P70S6K_pT389**	Stat3	-0.43	5.48E-23
**P70S6K_pT389**	RPA32	-0.417	1.59E-21
**P70S6K_pT389**	PKCPANBETAII_pS660	-0.415	2.47E-21
**P70S6K_pT389**	MRE11	0.414	2.99E-21
**P70S6K_pT389**	ERALPHA	0.413	4.46E-21
**P70S6K_pT389**	YTHDF2	-0.408	1.43E-20
**P70S6K_pT389**	SETD2	0.407	1.77E-20
**P70S6K_pT389**	RAD50	-0.406	2.05E-20
**P70S6K_pT389**	CYCLIND1	0.404	3.72E-20
**PEA15**	YAP_pS127	0.617	1.77E-51
**PEA15**	1433ZETA	0.565	1.04E-41
**PEA15**	p44-42-MAPK	0.473	4.50E-28
**PEA15**	DJ1	0.469	1.87E-27
**PEA15**	GSK3ALPHABETA	0.44	4.40E-24
**PEA15**	P90RSK_pT359S363	-0.435	1.74E-23
**PEA15**	CRAF_pS338	-0.427	1.27E-22
**PEA15**	S6	0.401	6.90E-20
**RRM2**	Myt1	0.623	1.16E-52
**RRM2**	RRM1	0.535	9.61E-37
**RRM2**	ATR_pS428	0.53	5.26E-36

**RRM2**	P27_pT198	0.518	3.42E-34
**RRM2**	Wee1_pS642	0.502	8.05E-32
**RRM2**	eIF4E_pS209	0.484	2.11E-29
**RRM2**	Twist	0.477	1.75E-28
**RRM2**	CYCLINB1	0.456	5.95E-26
**RRM2**	RIP3	0.439	6.04E-24
**RRM2**	DAPK2	0.437	1.04E-23
**RRM2**	PI3K-p110-b	0.423	3.72E-22
**RRM2**	c-IAP2	0.404	3.31E-20
**RRM2**	FOXM1	0.402	4.97E-20
**SHP2_pY542**	Pyk2_pY402	0.744	2.10E-85
**SHP2_pY542**	FAK_pY397	0.716	1.85E-76
**SHP2_pY542**	SHC_pY317	0.698	5.44E-71
**SHP2_pY542**	SRC_pY527	0.665	2.70E-62
**SHP2_pY542**	MAPK_pT202Y204	0.653	2.02E-59
**SHP2_pY542**	SRC_pY416	0.652	3.56E-59
**SHP2_pY542**	EGFR_pY1068	0.579	4.74E-44
**SHP2_pY542**	STAT3_pY705	0.548	7.94E-39
**SHP2_pY542**	IGF1R_pY1135Y1136	0.522	9.98E-35
**SHP2_pY542**	DDR1_pY513	0.495	7.36E-31
**SHP2_pY542**	HER3_pY1289	0.423	3.20E-22
**SHP2_pY542**	HER2_pY1248	0.409	1.06E-20
**SHP2_pY542**	FRS2-alpha_pY196	0.408	1.20E-20
**SHP2_pY542**	BETACATENIN	0.408	1.44E-20
**SHP2_pY542**	AKT_pS473	0.402	5.86E-20
**SHP2_pY542**	CDK1_pT14	-0.401	6.81E-20
**UGT1A**	Enolase-1	0.59	3.44E-46
**UGT1A**	HSP27_pS82	0.471	9.64E-28
**UGT1A**	VHL	-0.446	8.60E-25
**UGT1A**	PKM2	0.445	1.11E-24
**UGT1A**	IR-b	0.443	2.11E-24
**UGT1A**	SIRP-alpha	0.431	4.94E-23
**UGT1A**	MCT4	0.427	1.26E-22
**UGT1A**	Calnexin	0.413	4.25E-21
**UGT1A**	SOD2	0.408	1.43E-20
**UGT1A**	H2AX_pS140	0.402	4.90E-20
**UGT1A**	IRF-3_pS396	0.402	5.45E-20
**Vinculin**	TSC1	0.513	2.26E-33
**Vinculin**	RICTOR	0.507	1.24E-32
**Vinculin**	DNA-Ligase-IV	0.505	2.37E-32
**Vinculin**	Slfn11	0.485	1.47E-29
**Vinculin**	MYH11	0.419	8.82E-22
**Vinculin**	STING	0.418	1.30E-21
**Vinculin**	AKT	0.404	3.51E-20
